# Linguistic structure from a bottleneck on sequential information processing

**DOI:** 10.1038/s41562-025-02336-w

**Published:** 2025-11-24

**Authors:** Richard Futrell, Michael Hahn

**Affiliations:** 1 University of California, Irvine, Irvine, CA USA; 2 Saarland University, Saarbrücken, Germany

**Keywords:** Language and linguistics, Psychology, Information theory and computation

## Abstract

Human language has a distinct systematic structure, where utterances break into individually meaningful words that are combined to form phrases. Here we show that natural-language-like systematicity arises in codes that are constrained by a statistical measure of complexity called predictive information, also known as excess entropy. Predictive information is the mutual information between the past and future of a stochastic process. In simulations, we find that codes that minimize predictive information break messages into groups of approximately independent features that are expressed systematically and locally, corresponding to words and phrases. Next, drawing on cross-linguistic text corpora, we find that actual human languages are structured in a way that yields low predictive information compared with baselines at the levels of phonology, morphology, syntax and lexical semantics. Our results establish a link between the statistical and algebraic structure of language and reinforce the idea that these structures are shaped by communication under general cognitive constraints.

## Abstract (web summary)

Futrell and Hahn argue that languages can be understood as codes that minimize predictive information; that is, they express approximately independent features systematically and locally.

## Main

**Fig. 1:**
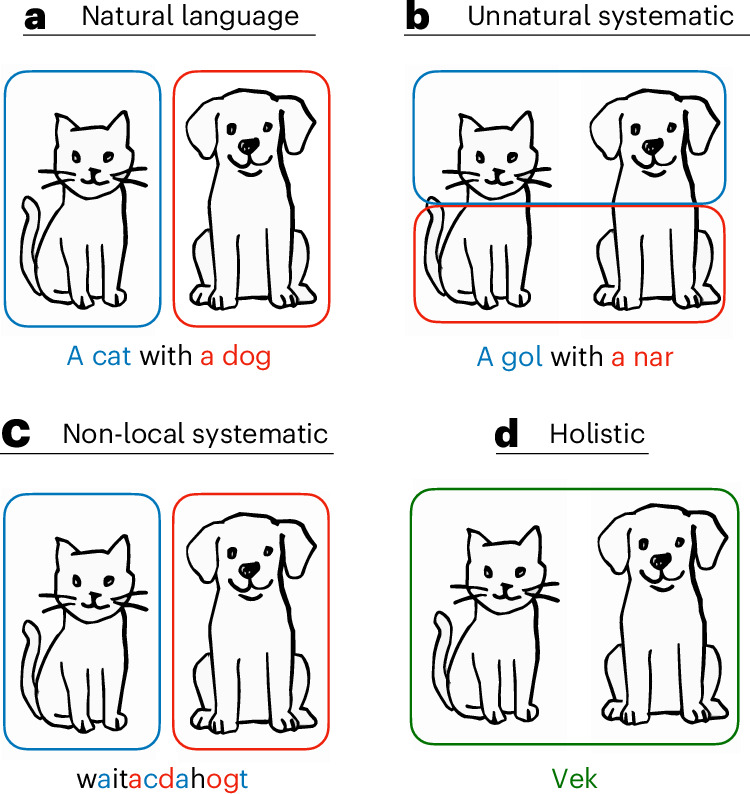
Example utterances describing an image in English and various hypothetical languages. **a**, An English utterance exhibiting natural local systematicity. **b**, An unnatural systematic language in which ‘gol’ means a cat head paired with a dog head and ‘nar’ means a cat body paired with a dog body. **c**, A non-local but systematic language in which an utterance is formed by interleaving the words for ‘cat’ and ‘dog’. **d**, A holistic language in which the form ‘vek’ means ‘a cat with a dog’ with no correspondence between parts of form and parts of meaning.

Human language is organized around a systematic, compositional correspondence between the structure of utterances and the structure of the meanings that they express^1^. For example, an English speaker will describe an image such as Fig. 1a with an utterance such as ‘a cat with a dog’, in which the parts of the the image correspond regularly with parts of the utterance such as ‘cat’—what we call words. This way of relating form and meaning may seem natural, but it is not logically necessary. For example, Fig. 1b shows an utterance in a hypothetical counterfactual language where meaning is decomposed in a way that most people would find unnatural: here, we have a word ‘gol’, which refers to a cat head and a dog head together, and another word ‘nar’, which refers to a cat body and a dog body together. Similarly, Fig. 1c presents a hypothetical language that is systematic but with an unnatural way of decomposing the utterance: here, the utterance contains individually meaningful subsequences ‘a cat’, ‘with‘ and ‘a dog’, but these are interleaved together, rather than concatenated as they are in English. We can even conceive of languages such as in Fig. 1d, where each meaning is expressed holistically as a single unanalysable form^2,3^—in fact, this lack of systematic structure is expected in optimal codes like Huffman codes^4,5^. Why is human language the way it is, and not like these counterfactuals?

We argue that the particular structure of human language can be derived from general constraints on sequential information processing. We start from three observations:

1. Utterances consist, to a first approximation, of one-dimensional sequences of discrete symbols (for example, phonemes).
2. The ease of production and comprehension of these utterances is influenced by the sequential predictability of these symbols down to the smallest timescales^6–11^.
3. Humans have limited cognitive resources for use in sequential prediction^12–16^.

Thus, we posit that language is structured in a way that minimizes the complexity of sequential prediction, as measured using a quantity called predictive information: the amount of information about the past of a sequence that any predictor must use to predict its future^17,18^, also called excess entropy^19,20^. Below, we find that codes that are constrained to have low predictive information within signals have systematic structure resembling natural language, and we provide massively cross-linguistic empirical evidence based on large text corpora showing that natural language has lower predictive information than would be expected if it had different kinds of structure.

## Results

### Explananda

**Fig. 2:**
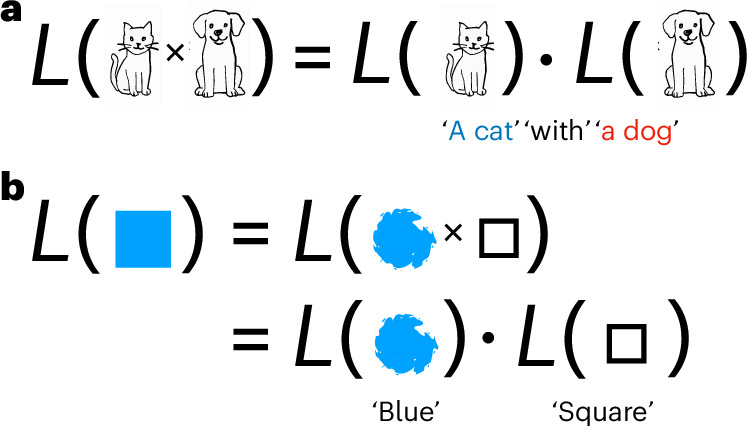
Two examples of linguistic systematicity as a homomorphism. *L*(⋅) stands for the English language, seen as a function from meanings to forms (strings). **a**, The meaning naturally decomposes into two features corresponding to the two animals. The form ‘a cat with a dog’ decomposes systematically into forms for the cat and the dog, concatenated together with the string ‘with’ between them. **b**, The meaning naturally decomposes into two features, corresponding to colour and shape. The form ‘blue square’ decomposes systematically into forms for the colour and the shape, concatenated together.

First, we clarify what we want to explain. Taking a maximally general stance, we think of a language as a function mapping meanings to forms, where meanings are any objects in a set ${\mathcal{M}}$, and forms are strings drawn from a finite alphabet of letters *Σ*, typically standing for phonemes. We say a language is systematic when it is a homomorphism^21,22^, as illustrated in Fig. 2. That is, if a meaning *m* can be decomposed into parts (say *m* = *m*_1_ × *m*_2_), then the string for that meaning decomposes in the same way:

1 $$L({m}_{1}\times {m}_{2})=L({m}_{1})\cdot L({m}_{2}),$$

where ‘⋅’ is some means of combining two strings, such as concatenation. For example, an object  would be described in English as *L*() = blue square. The meaning  is decomposed into features for colour and shape, and these features are expressed systematically as the words ‘blue’ and ‘square’ concatenated together.

We wish to explain why human languages are systematic, why they decompose meanings in the way they do, and why they combine strings in the way they do. In particular, meanings are decomposed in a way that seems natural to humans (that is, like Fig. 1a and not Fig. 1b), a property we call ‘naturalness’. Also, strings are usually combined by concatenation (that is, like Fig. 1a and not like Fig. 1c), or more generally by some process that keeps relevant parts of the string relatively close together. We call this property ‘locality’.

Influential accounts have held that human language is systematic because language learners need to generalize to produce forms for never-before-seen meanings^23–26^. Such accounts successfully motivate systematicity in the abstract sense, but on their own they do not explain naturalness and locality. However, a theory of systematicity must have something to say about these properties, because if we are free to choose any arbitrary functions ‘×’ and ‘⋅’, then any function *L* can be considered systematic in the sense of equation (1), and the idea of systematicity becomes vacuous^27^.

In existing work, naturalness and locality are explained via (implicit or explicit) inductive biases built into language learners^23,28–35^ or stipulations about the mental representation or perception of meanings^36–40^. By contrast, we aim to explain natural local systematicity in language from maximally general principles, without any assumptions about the mental representation of meaning, and with extremely minimal assumptions about the structure of forms—only that they are ultimately expressed as one-dimensional sequences of discrete symbols.

### Predictive Information

We measure the complexity of sequential prediction using predictive information, which is the amount of information that any predictor must use about the past of a stochastic process to predict its future (below, we assume familiarity with information-theoretic quantities of entropy and mutual information^41^). Given a stationary stochastic process generating a stream of symbols …, *X*_*t*−1_, *X*_*t*_, *X*_*t*+1_, …, we split it into ‘the past’ *X*_past_, representing all symbols up to time *t*, and ‘the future’ ${X}_{{\rm{future}}}$, representing all symbols at time *t* or after. The predictive information or excess entropy^18,19^ *E* is the mutual information between the past and the future:

2 $$E={{\rm{I}}}[{X}_{{\rm{past}}}:{X}_{{\rm{future}}}].$$

We calculate the predictive information of a language *L* as the predictive information of the stream of letters generated by repeatedly sampling meanings $m\in {\mathcal{M}}$ from a source distribution, translating them to strings as *s* = *L*(*m*) and concatenating them with a delimiter in between.

Predictive information can be calculated in a simple way that gives intuition about its behaviour. Let *h*_*n*_ represent the *n*-gram entropy of a process, that is, the average entropy of a symbol given a window of *n* − 1 previous symbols:

3 $${h}_{n}={{\rm{H}}}[{X}_{t}| {X}_{t-n+1},\ldots ,{X}_{t-1}].$$

**Fig. 3:**
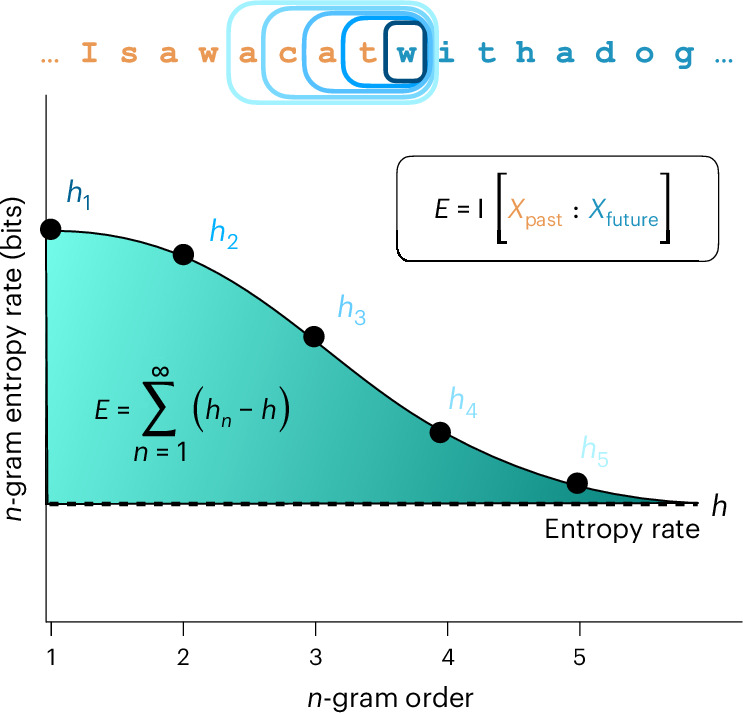
Schematic calculation of predictive information as the sum of *n*-gram entropies *h*_*n*_ minus the asymptotic entropy rate *h*.

As the window size increases, the *n*-gram entropy decreases to an asymptotic value called the entropy rate *h*. The predictive information represents the convergence to the entropy rate,

4 $$E=\mathop{\sum }\limits_{n=1}^{\infty }\left({h}_{n}-h\right),$$

as illustrated in Fig. 3. This calculation reveals that predictive information is low when symbols can be predicted accurately on the basis of local contexts, that is, when *h*_*n*_ is close to *h* for small *n*.

### Simulations

The following simulations show that, when languages minimize predictive information, they express approximately independent features systematically and locally in a way that corresponds to words and phrases in natural language.

#### Systematic expression of independent features

**Fig. 4:**
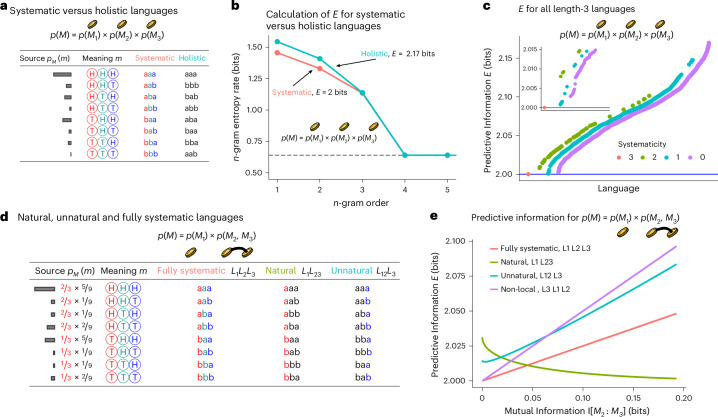
Simulations of languages for coin-flip distributions. **a**, Two unambiguous languages for meanings consisting of three weighted coin flips. In the systematic language, each letter corresponds to the outcome from one coin flip. In the holistic language, there is no natural systematic relationship between the form and the meaning. **b**, Calculation of predictive information for the source and two languages in **a**. The systematic language has lower predictive information. **c**, Predictive information of all bijective mappings from meanings to length-3 binary strings, for the meanings and source in **a**. Languages are ordered by predictive information and coloured by the number of coin flips expressed systematically: 3 for a fully systematic language and 0 for a fully holistic language. The inset box zooms in on the region of low predictive information. **d**, Languages used in **e** along with an example source, which has mutual information I[*M*_2_: *M*_3_] ≈ 0.18 bits. **e**, Predictive information of various languages for varying levels of mutual information between coin flips *M*_2_ and *M*_3_ (see text). Zero mutual information corresponds to **b** and **c**. The ‘natural’ language expresses *M*_2_ and *M*_3_ together holistically. The ‘unnatural’ language expresses *M*_1_ and *M*_2_ together holistically.

Consider a set of meanings consisting of the outcomes of three weighted coin flips. In a natural systematic language, we would expect each string to have contiguous ‘words’ corresponding to the outcome of each individual coin, whereas a holistic language would have no such structure, as shown in the examples in Fig. 4a. It turns out that, for these example languages, the natural systematic one has lower predictive information, as shown in Fig. 4b. In fact, among all possible unambiguous length-3 binary languages, predictive information is minimized exclusively in the systematic languages, as shown in Fig. 4c.

Intuitively, the reason systematic languages minimize predictive information here is that the features of meaning expressed in each individual letter are independent of each other, and so there is no statistical dependence among letters in the string. The general pattern is that an unambiguous language that minimizes predictive information will find features that have minimal mutual information and express them systematically. See Supplementary Section A for formal arguments to this effect.

#### Holistic expression of correlated components

What happens to predictive information when the source distribution cannot be expressed in terms of fully independent features? In that case, it is better to express the more correlated features holistically, without systematic structure. This holistic mapping is what we find in natural language for individual words (or, more precisely, morphemes), according to the principle of arbitrariness of the sign^42^. For example, the word ‘cat’ has no identifiable parts that systematically correspond to features of its meaning. Furthermore, as we will discuss below, morphemes in language typically encode categories whose semantic features are highly correlated with each other^43^.

We demonstrate this effect in simulations by varying the coin-flip scenario above. Denote the three coin flips as *M*_1_, *M*_2_ and *M*_3_. Imagine the second and third coins and are tied together, so that their outcomes *M*_2_ and *M*_3_ are correlated, as in the example in Fig. 4d. In the limit where *M*_2_ and *M*_3_ are fully correlated, these coin flips have effectively become one feature. Figure 4e shows predictive information for a number of possible languages in this setting, as a function of the mutual information between the tied coin flips *M*_2_ and *M*_3_. In the low-mutual-information regime—where *M*_2_ and *M*_3_ are nearly independent—the best language is still fully systematic. However, as mutual information increases, the best language is one that expresses the tied coin flips *M*_2_ and *M*_3_ together holistically, as a single ‘word’. An unnatural language that expresses the uncorrelated coin flips *M*_1_ and *M*_2_ holistically is much worse, as is a non-local systematic language that breaks up the ‘word’ corresponding to the correlated coin flips *M*_2_ and *M*_3_.

#### Locality

Next, we show that minimization of predictive information yields languages where features of meaning correspond to localized parts of strings, corresponding to words. We consider a Zipfian distribution over 100 meanings, and a language *L* in which forms consist of two length-4 ‘words’. We then consider scrambled languages formed by applying permutations to the string output of *L*. For example, if the original language expresses a meaning with two words such as *L*(*m*_1_ × *m*_2_) = aaaa ⋅ bbbb, a possible scrambled language would have ${L}^{{\prime} }({m}_{1}\times {m}_{2})={\mathtt{baaabbab}}$. These scrambled languages instantiate possible string combination functions other than concatenation.

**Fig. 5:**
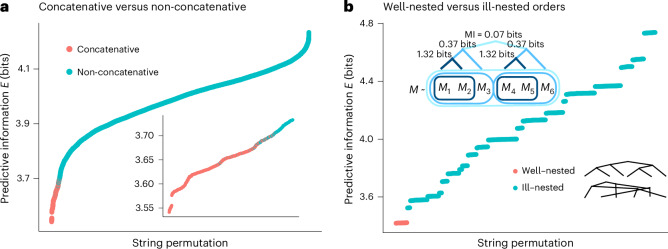
Simulations of codes with different orders of elements. **a**, Predictive information of all string permutations of a systematic language for a Zipfian source. Languages that combine components by concatenation, marked in red, achieve the lowest predictive information. The inset zooms in on the 2,000 permutations with the lowest predictive information. **b**, A hierarchically structured source distribution (see text) and predictive information of all permutations of a systematic language for this source. A language is well nested when all groups of letters corresponding to groupings in the inset tree figure are contiguous. The well-nested languages achieve the lowest predictive information.

Calculating predictive information for all possible scrambled languages, we find that the languages in which the ‘words’ remain contiguous have the lowest predictive information, as shown in Fig. 5a. This happens because the coding procedure above creates correlations among letters within a word. When these correlated letters are separated from each other—such as when letters from another word intervene—then predictive information increases. Interestingly, not every concatenative language is better than every non-concatenative one. This corresponds to the reality of natural language, in which limited non-concatenative and non-local morphophonological processes do exist, for example, in Semitic non-concatenative morphology^44^.

#### Hierarchical structure

Natural language sentences typically have well-nested hierarchical syntactic structures, of the kind generated by a context-free grammar^45^: for example, the sentence ‘[[the big dog] chased [a small cat]]’ has two noun phrases, indicated by brackets, which are contiguous and nested within the sentence. Minimization of predictive information creates these well-nested word orders, with phrases corresponding to groups of words that are more or less strongly correlated^46^. We demonstrate this effect using a source distribution defined over six random variables *M*_1_, …, *M*_6_ with a covariance structure shown in the inset of Fig. 5b: each of the variable pairs (*M*_1_, *M*_2_) and (*M*_4_, *M*_5_) are highly internally correlated; these pairs are weakly correlated with *M*_3_ and *M*_6_, respectively; and both groups of variables are very weakly correlated with each other. As above, we consider all possible permutations of a systematic code for these source variables. The codes that minimize predictive information are those that are well nested with respect to the correlation structure of the source, keeping the letters corresponding to all groups of correlated features contiguous. Further simulation results involving context-free languages are found in Supplementary Section G. For a mathematical analysis of predictive information in local and random orders for structured sources, see Supplementary Section A.

### Cross-linguistic empirical results

Here, we present cross-linguistic empirical evidence that the systematic structure of language has the effect of reducing predictive information at the levels of phonotactics, morphology, syntax and semantics, compared against systems that lack natural local systematicity.

#### Phonotactics

**Fig. 6:**
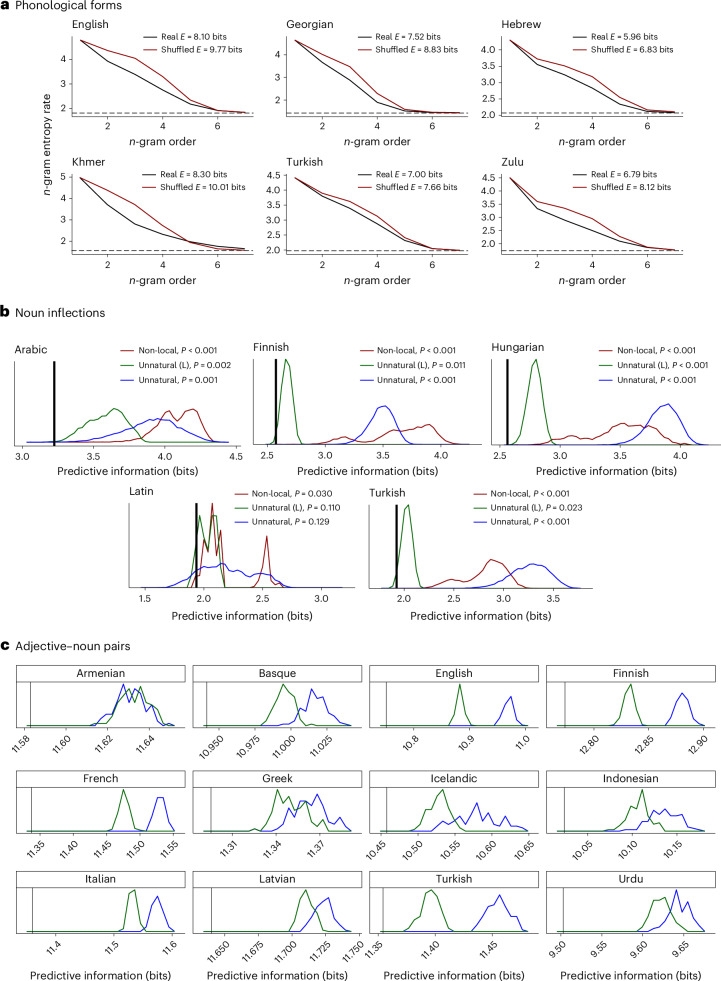
Evidence that natural languages are configured in a way that reduces predictive information, in phonotactics, morphology and syntax. **a**, Predictive information calculation for phonological forms in selected languages, comparing the attested forms against forms that have been deterministically shuffled while preserving manner of articulation. **b**, Letter-level predictive information of noun morphology (vertical black line), compared against predictive information values for four random baselines (densities of 10,000 samples; see text). *P* values indicate the proportion of baseline samples with lower predictive information than the attested forms. **c**, Letter-level predictive information of adjective–noun pairs from 12 languages, compared with baselines. Non-local baselines always generate much higher predictive information than the attested forms and are not shown.

Languages have restrictions on what sequences of sounds may occur within words: for example, ‘blick’ seems like a possible English word, whereas ‘bnick’ does not, even though it is pronounceable in other languages^47^. These systems of restrictions are called phonotactics. Here, we show that actual phonotactic systems of human languages, which involve primarily local constraints on what sounds may co-occur, result in lower predictive information compared with counterfactual phonotactic systems. We compare phonemically transcribed wordforms in vocabulary lists of 61 languages against counterfactual alternatives generated by deterministically scrambling phonemes within a word while preserving manner of articulation. This ensures that the resulting counterfactual forms are roughly possible to articulate. For example, an English word ‘fasted’ might be scrambled to form ‘sefdat’. Calculating predictive information, we find that the real vocabulary lists have lower predictive information than the counterfactual variants in all languages tested. Results for six languages with diverse sound systems are shown in Fig. 6a. Results for the remaining 55 languages are presented in Supplementary Section C.

#### Morphology

**Fig. 7:**
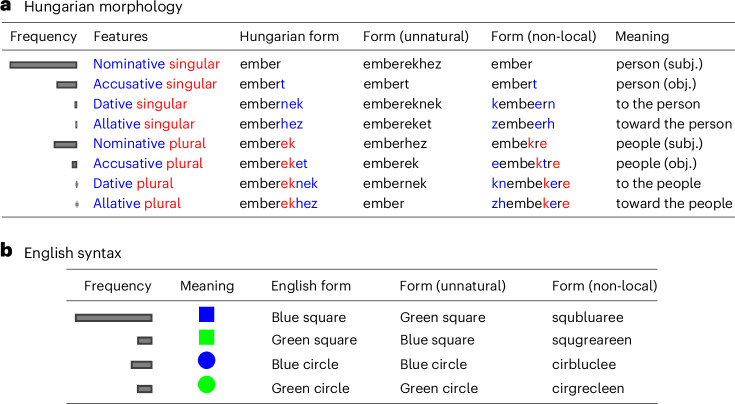
Examples of systematic morphology and syntax, and baselines used in experiments. **a**, Forms of the Hungarian noun ‘ember’ meaning ‘person’, along with examples of the unnatural and non-local baseline used in Fig. 6b. An additional 231 forms are not shown. The ‘Frequency’ column illustrates the joint frequency of grammatical features in the Hungarian Szeged UD corpus^100,106^. **b**, English forms for the given meanings, along with frequencies from the English Common Crawl web corpus^107^. Example unnatural and non-local baseline forms are shown.

Words change form to express grammatical features in a way that is often systematic. For example, the forms of the Hungarian noun shown in Fig. 7a are locally systematic with respect to case and number features. In Fig. 6b, we show that the local systematic structure of affixes for case, number, possession and definiteness in five languages has the effect of reducing predictive information when comparing against baselines that disrupt this structure. We estimate predictive information of these morphological affixes across five languages, with source distributions proportional to empirical corpus counts of the joint frequencies of grammatical features. We compare the predictive information of the attested forms against three alternatives: (1) a non-local baseline generated by applying a deterministic permutation function to each form, (2) an unnatural baseline generated by permuting the assignment of forms to meanings (features) and (3) a more controlled unnatural baseline that permutes the form–meaning mapping while preserving form length. The unnatural baselines preserve the phonotactics of the original forms; only the form–meaning relationship is changed. We generate 10,000 samples (permutations) for each of the three baselines per language.

Across the languages, we find that the attested forms have lower predictive information than the majority of samples of the baselines. The weakest effect is in Latin, which also has the most fusional and least systematic morphology^48^. Note that Arabic nouns often show non-concatenative morphology in the form of so-called broken plurals: for example, the plural of the loanword ‘film’ meaning ‘film’ is *’aflām*. This pattern is represented in the forms used to generate Fig. 6b, and yet Arabic noun forms still have lower predictive information than the majority of baseline samples. This suggests that the limited form of non-concatenative morphology present in Arabic is still consistent with the idea that languages are configured in a way that keeps predictive information low.

#### Syntax

Phrases such as ‘blue square’ have natural local systematicity, as shown in Fig. 7b. We compare real adjective–noun combinations in corpora of 12 languages against unnatural and non-local baselines generated the same way as in the morphology study: permuting the letters within a form to disrupt locality, or permuting the assignment of forms to meanings to disrupt naturalness. We estimate the probability of a meaning as proportional to the frequency of the corresponding adjective–noun pair. Results are shown in Fig. 6c. The real adjective–noun pairs have lower predictive information than a large majority of baselines across all languages tested.

#### Word order

**Fig. 8:**
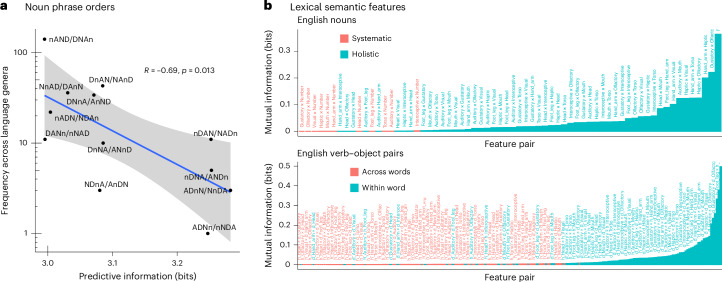
Evidence that word order and lexical semantics are configured in ways that reduce predictive information. **a**, Typological frequency of noun phrase orders (number of unrelated language genera showing the given order^49^) as a function of predictive information. More frequent orders have lower predictive information. The blue line shows a linear regression predicting log typological frequency from predictive information. Error bars indicate a 95% confidence interval of the slope of this regression. The negative correlation is significant with Pearson’s *R* = −0.69 and *P* = 0.013. **b**, Top: pairwise mutual information of semantic features from the Lancaster Sensorimotor Norms^53^ in addition to a number feature, as indicated by plural morphology. The number feature is expressed systematically; all others are holistic. Bottom: pairwise mutual information values for Lancaster Sensorimotor Norm features across and within words, for pairs of verbs and their objects.

In an English noun phrase such as ‘the three cute cats’, the elements Determiner (D, ‘the’), Numeral (N, ‘three’), Adjective (A, ‘cute’) and Noun (n, ‘cats’) are combined in the order D–N–A–n. This order varies across languages—for example, Spanish has D–N–n–A (‘los tres gatos lindos’)—but certain orders are more common than others^49^. We aim to explain the cross-linguistic distribution of these orders through reduction of predictive information, which drives words that are statistically predictive of each other to be close to each other, an intuition shared with existing models of adjective order^40,46,50^. To do so, we estimate source probabilities for noun phrases (consisting of single head lemmas for a noun along with an optional adjective, numeral and determiner) based on corpus frequencies. We then calculate predictive information at the word level (treating words as single atomic symbols) for all possible permutations of D–N–A–n. Predictive information is symmetric with respect to time reversal, so we cannot distinguish orders such as D–N–A–n from n–A–N–D and so on. As shown in Fig. 8a, the orders with lower predictive information are also the orders that are more frequent cross-linguistically. A number of alternative source distributions also yield this downward correlation, as shown in Supplementary Section D.

#### Lexical semantics

Considering a word such as ‘cats’, all the semantic features of a cat (furriness, mammalianness and so on) are expressed holistically in the morpheme ‘cat’, while the feature of numerosity is separated into the plural marker ‘–s’. Plural marking like this is common across languages^51,52^. From reduction of predictive information, we expect relatively uncorrelated components of meaning to be expressed systematically, and relatively correlated components to be expressed together holistically. Thus, we hold that numerosity is selected to be expressed systematically in a separate morpheme because it is relatively independent of the other features of nouns, which are in turn highly correlated with each other. Our theory thus derives the intuition that natural categories arise from the correlational structure of experience^43^.

We validate this prediction in a study of semantic features in English, using the Lancaster Sensorimotor Norms^53^ to provide semantic features for English words and using the English Universal Dependencies (UD) corpus to provide a frequency distribution over words. The Lancaster Sensorimotor Norms provide human ratings for words based on sensorimotor dimensions, such as whether they involve the head or arms. As shown in Fig. 8b (top), we find that the semantic norm features are highly correlated with each other, and relatively uncorrelated with numerosity, as predicted by the theory.

For the same reason, the theory also predicts that semantic features should be more correlated within words than across words. In Fig. 8b (bottom), we show within-word and cross-word correlations of the semantic norm features for pairs of verbs and their objects taken from the English UD corpus. As predicted, the across-word correlations are weaker. Correlations based on features drawn from other semantic norms are presented in Supplementary Section E.

## Discussion

Our results underscore the fundamental roles of prediction and memory in human cognition and provide a link between the algebraic structure of human language and information-theoretic concepts used in machine learning and neuroscience. Our work joins the growing body of information-theoretic models of human language based on resource-rational efficiency^54–59^.

### Language models

Large language models are based on neural networks trained to predict the next token of text given previous tokens. Our results suggest that language is structured in a way that makes this next-token prediction relatively easy, by minimizing the amount of information that needs to be extracted from the previous tokens to predict the following tokens. Although it has been claimed that large language models have little to tell us about the structure of human language—because their architectures do not reflect formal properties of grammars and because they can putatively learn unnatural languages as well as natural ones^60–62^—our results suggest that these models have succeeded so well precisely because natural language is structured in a way that makes their prediction task relatively simple. Indeed, neural sequence architectures struggle to learn languages that lack information locality^63,64^.

### Machine learning

Our results establish a connection between the structure of human language and ideas from machine learning. In particular, minimization of mutual information (a technique known as independent components analysis, ICA^65,66^) is widely deployed to create representations that are ‘disentangled’ or compositional^67^, and to detect object boundaries in images, under the assumption that pixels belonging to the same object exhibit higher statistical dependence than pixels belonging to different objects^68^. (Although general nonlinear ICA with real-valued outputs does not yield unique solutions^69^, we have found above that minimization of predictive information does find useful structure in our setting, with discrete string-valued outputs and a deterministic function mapping meaning to form.) We propose that human language follows a similar principle: it reduces predictive information, which amounts to performing a generalized sequential ICA on the source distribution on meanings, factoring it into groups of relatively independent components that are expressed systematically as words and phrases, with more statistical dependence within these units than across them. This provides an explanation for why ICA-like objectives yield representations that are intuitively disentangled, compositional, or interpretable: they yield the same kinds of concepts that we find encoded in natural language.

### Neuroscience

Similarly, neural codes have been characterized as maximizing information throughput subject to information-theoretic and physiological constraints^70,71^, including explicit constraints on predictive information^72,73^. These models predict that, in many cases, neural codes are decorrelated: distinct neural populations encode statistically independent components of sensory input^74^. Our results suggest that language operates on similar principles: it expresses meanings in a way that is temporally decorrelated. This view is compatible with neuroscientific evidence on language processing: minimization of predictive information (while holding overall predictability constant) equates to maximization of local predictability of the linguistic signal, a driver of the neural response to language^10,75^.

### Information theory and language

Previous work^76^ derived locality in natural language from a related information-theoretic concept, the memory–surprisal trade-off or predictive information bottleneck curve, which describes the best achievable sequential predictability as a function of memory usage^77^. The current theory is a simplification that looks at only one part of the curve: predictive information is the minimal memory at which sequential predictability is maximized. A more complete information-theoretic view of language may have to consider the whole curve.

We join existing work attempting to explain linguistic structure on the basis of information-theoretic analysis of language as a stochastic process, for example, the study of lexical scaling laws as a function of redundancy and non-ergodicity in text^78^. Other work on predictive information in language has focused on the long-range scaling of the *n*-gram entropy in connected texts, with results seeming to imply that the predictive information diverges^79,80^. By contrast, we have focused on only single utterances, effectively considering only relatively short-range predictive information.

### Cognitive status of predictive information

Predictive information is a fundamental measure of complexity, which may manifest explicitly or implicitly in various ways in the actual mechanisms of language production, comprehension and learning. For example, in a recent model of online language comprehension^81^, comprehenders predict upcoming words on the basis of memory representations that are constrained to store only a small number of words. The fundamental limits of predictive information apply implicitly in this model because comprehenders’ predictions cannot be more accurate than if they stored an equivalent amount of predictive information. As another example, a model of language production based on short stored chunks^46^ would effectively produce language with low predictive information, because these chunks would be relatively independent of each other, while predictive relationships inside the stored chunks would be preserved. Predictive information has also been linked to difficulty of learning: processes containing more predictive information require more parameters and data to be learned^18^, and any learner with limited ability to learn long-term dependencies will have an effective inductive bias towards languages with low predictive information. Predictive information is not meant as a complete model of the constraints on language, which would certainly involve factors beyond predictive information as well as separate, potentially competing pressures from comprehension and production^82^.

Relatedly, while we have shown that natural language is configured in a way that keeps predictive information low, we have not speculated on how languages come to be configured in this way, in terms of language evolution and change. We believe there are multiple pathways for this to happen. For example, efficiency pressures in individual interactions could give rise to overall efficient conventions^83^, or memory limits in learning^84,85^ could cause learners to form low-predictive-information generalizations from their input. Identifying the causal mechanisms that control predictive information in language is a critical topic for future work.

### Linguistics

Our theory of linguistic systematicity is independent of theoretical assumptions about mental representations of grammars, linguistic forms or the meanings expressed in language. Predictive information is a function only of the probability distribution on forms, seen as one-dimensional sequences of symbols unfolding in time. This independence from representational assumptions is an advantage, because there is as yet no consensus about the basic nature of the mental representations underlying human language^86,87^.

Our results reflect and formalize a widespread intuition about human language, first formulated as Behaghel’s Law^88^: ‘that which is mentally closely related is also placed close together’. For example, words are contiguous units and the order of morphemes within them is determined by a principle of relevance^89,90^, and important aspects of word order across languages have been explained in terms of dependency locality, the principle that syntactically linked words are close^91–94^.

A constraint on predictive information predicts information locality: elements of a linguistic form should be close to each other when they predict each other^50^. We propose that information locality subsumes existing intuitive locality ideas. Thus, because words have a high level of statistical interpredictability among their parts^95^, they are mostly contiguous, and as a residual effect of this binding force, related words are also close together. Furthermore, we have found that the same formal principle predicts the existence of linguistic systematicity and the way that languages divide the world into natural kinds^37,43^.

### Limitations

Much work is required to push our hypothesis to its limit. We have assumed throughout that languages are one-to-one mappings between form and meaning; the behaviour of ambiguous or non-deterministic codes, where ambiguity might trade off with predictive information, may yield additional insight. Furthermore, we have examined predictive information only within isolated utterances. It remains to be seen whether reduction of predictive information, applied at the level of many connected utterances, would be able to explain aspects of discourse structure such as the hierarchical organization of topics and topic–focus structure^96^.

One known limitation of our theory is that predictive information is symmetric with respect to time reversal, so (at least when applied at the utterance level) it cannot explain time-asymmetric properties of language such as the pattern of ‘accessible’ (frequent, animate, definite and given) words appearing earlier within utterances than inaccessible ones^97,98^. There is also the fact that non-local and non-concatenative structures do exist in language, for example, long-term coreference relationships among discourse entities, and long-distance filler–gap dependencies, which would seem to contravene the idea that predictive information is constrained. An important area for future research will be to determine what effect these structures really have on predictive information, and what other constraints on language might explain them.

## Methods

### Constructing a stochastic process from a language

We define a language as a mapping from a set of meanings to a set of strings, $L:{\mathcal{M}}\to {\Sigma }^{* }$. To define predictive information of a language, we need a way to derive a stationary stochastic process generated by that language. We use the following mathematical construction that generates an infinite stream of symbols: (1) meanings *m* ~ *p*_*M*_ are sampled i.i.d. from the source distribution *p*_*M*_, (2) each meaning is translated into a string as *s* = *L*(*m*), and (3) the strings *s* are concatenated end-to-end in both directions with a delimiter *#* ∉ *Σ* between them. Finally, a string is chosen with probability reweighted by its length, and a time index *t* (relative to the closest delimiter to the left) is selected uniformly at random within this form.

This construction has the effect of zeroing out any mutual information between symbols with the delimiter between them. Thus, when we compute *n*-gram statistics, we can treat each form as having infinite padding symbols to the left and right. This is the standard method for collecting *n*-gram statistics in natural language processing^99^.

### Three-feature source simulation

For Fig. 4b,c, the source distribution is distributed as a product of three Bernoulli distributions:

5 $$M \sim {\rm{Bernoulli}}\left(\frac{2}{3}\right)\times {\rm{Bernoulli}}\left(\frac{2}{3}+\varepsilon \right)\times {\rm{Bernoulli}}\left(\frac{2}{3}+2\varepsilon \right),$$

with *ε* = 0.05.

For Fig. 4e, we need to generate distributions of the form *p*(*M*) = *p*(*M*_1_) × *p*(*M*_2_, *M*_3_) while varying the mutual information I[*M*_2_: *M*_3_]. We start with the source from equation (5) (whose components are here denoted *p*_indep_) and mix it with a source that creates a correlation between *M*_2_ and *M*_3_:

6 $$\begin{array}{l}{p}_{\alpha }(M=ijk)={p}_{{\rm{indep}}}({M}_{1}=i)\\\times \left[\left(1-\alpha \right)\left({p}_{{\rm{indep}}}({M}_{2}=j)\times {p}_{{\rm{indep}}}({M}_{3}=k)\right)+\frac{\alpha }{2}{\delta }_{jk}\right],\end{array}$$

with *δ*_*jk*_ = 1 if *j* = *k* and 0 otherwise. The mixture weight *α* controls the level of mutual information, ranging from 0 at *α* = 0 to at most 1 bit at *α* = 1. A more comprehensive study of the relationship between feature correlation, systematicity and predictive information is given in Supplementary Section B, which examines systematic and holistic codes for a comprehensive grid of possible distributions on the simplex over four outcomes.

### Locality simulation

For the simulation shown in Fig. 5a, we consider a source over 100 objects labelled {*m*^00^, *m*^01^, …, *m*^99^}, following a Zipfian distribution $p(M={m}^{i})\propto {\left(i+1\right)}^{-1}$. We consider a language based on a decomposition of the meanings based on the digits of their index, with for example *m*^89^ decomposing into features as ${m}_{1}^{8}\times {m}_{2}^{9}$. Each utterance decomposes into two ‘words’ as *L*(*m*_1_ × *m*_2_) = *L*(*m*_1_) ⋅ *L*(*m*_2_), where the word for each feature *m*^*k*^ is a random string in {0, 1}^4^, maintaining a one-to-one mapping between features *m*^*k*^ and words.

### Hierarchy simulation

For the simulation shown in Fig. 5b, we consider a source *M* over 5^6^ = 15,625 meanings, which may be expressed in terms of six random variables $\left\langle {M}_{1},{M}_{2},{M}_{3},{M}_{4},{M}_{5},{M}_{6}\right\rangle$ each over five outcomes, with a probability distribution as follows:

7 $$\begin{array}{lll}p(M)\,=\,\alpha q({M}_{1},{M}_{2},{M}_{3},{M}_{4},{M}_{5},{M}_{6})+\left(1-\alpha \right)\\\,\,\left(\left[\beta q({M}_{1},{M}_{2},{M}_{3})+\left(1-\beta \right)\left[\gamma q({M}_{1},{M}_{2})+\left(1-\gamma \right)q({M}_{1})q({M}_{2})\right]q({M}_{3})\right]\right.\\\qquad\quad\times \left[\beta q({M}_{4},{M}_{5},{M}_{6})+\left(1-\beta \right)\right.\\\qquad\quad\left[\gamma\!\left.\left.q({M}_{4},{M}_{5})+\left(1-\gamma\right)q({M}_{4})q({M}_{5})\right]q({M}_{6})\right]\right),\end{array}$$

where *α* = 0.01, *β* = 0.20 and *γ* = 0.99 are coupling constants, and each *q*(⋅) is a Zipfian distribution as above. The coupling constants control the strengths of the correlations shown in Fig. 5b.

### Phonotactics

We assume a uniform distribution over forms found in WOLEX. Supplementary Section F shows results for four languages using corpus-based word frequency estimates to form the source distribution, with similar results.

### Morphology

We estimate the source distribution on grammatical features (number, case, possessor and definiteness) using the feature annotations from UD corpora, summing over all nouns, with add-1/2 smoothing. The dependency treebanks are drawn from UD v2.8^100^: for Arabic, NYUAD Arabic UD Treebank; for Finnish, Turku Dependency Treebank; for Turkish, Turkish Penn Treebank; for Latin, Index Thomisticus Treebank; for Hungarian, Szeged Dependency Treebank. Forms are represented with a dummy symbol ‘X’ standing for the stem, and then orthographic forms for suffixes, such as ‘Xoknak’ for the Hungarian dative plural. For Hungarian, Finnish and Turkish, we use the forms corresponding to back unrounded vowel harmony. For Latin, we use first-declension forms. For Arabic, we use regular masculine triptote forms with a broken plural; to do so, we represent the root using three dummy symbols, and the plural using a common ‘broken’ form^101^, with, for example, ‘XaYZun’ for the nominative indefinite singular and ‘’aXYāZun’ for the nominative indefinite plural. Results using an alternate broken plural form ‘XiYāZun’ are nearly identical.

### Adjective–noun pairs

From UD corpora, we extract adjective–noun pairs, defined as a head wordform with part-of-speech ‘NOUN’ modified by an adjacent dependent wordform with relation ‘amod’ and part-of-speech ‘ADJ’. The forms over which predictive information is computed consist of the pair of adjective and noun from the corpus, in their original order, in original orthographic form with a whitespace between them. The source distribution is directly proportional to the frequencies of the forms.

### Noun phrase order

The source distribution on noun phrases is estimated from the empirical frequency of noun phrases in the German GSD UD corpus, which has the largest number of such noun phrases among the UD corpora. To estimate this source, we define a noun phrase as a head lemma of part-of-speech ‘NOUN’ along with the head lemmas for all dependents of type ‘amod’ (with part-of-speech ‘ADJ’), ‘nummod’ (with part-of-speech ‘NUM’) and ‘det’ (with part-of-speech ‘DET’). We extract these noun phrase forms from the corpus. When a noun phrase has multiple adjectives, one of the adjectives is chosen randomly and the others are discarded. The result is counts of noun phrases of the form below:

**Table:** 

Determiner	Numeral	Adjective	Noun	Count
die	—	—	Hand	234
ein	—	alt	Kind	4
—	drei	—	Buch	2
ein	—	einzigartig	Parfümeur	1
…	…	…	…	…

The source distribution is directly proportional to these counts. We then compute predictive information at the word level over the attested noun phrases for all possible permutations of determiner, numeral, adjective and noun. Typological frequencies are as given by ref. ^49^.

### Semantic features

We binarize the Lancaster Sensorimotor Norms^53^ by recoding each norm as 1 if it exceeds the mean value for that feature across all words, and 0 otherwise. Word frequencies are calculated by maximum likelihood based on lemma frequencies in the concatenation of the English GUM^102^, GUMReddit^103^ and EWT^104^ corpora from UD 2.8. The ‘Number’ feature is calculated based on the value of the ‘Number’ feature in the UD annotations. Verb–object pairs were identified as a head wordform with part-of-speech ‘VERB’ with a dependent wordform of relation ‘obj’ and part-of-speech ‘NOUN’.

### Reporting summary

Further information on research design is available in the Nature Portfolio Reporting Summary linked to this article.

## Supplementary information

Supplementary Information Supplementary Sections A–G, Figs. 1–14, Tables 1 and 2 and References.

Reporting summary

## Data Availability

Unique data required to reproduce our results are available via GitHub at http://github.com/Futrell/infolocality. Corpus count data are drawn from Universal Dependencies v2.8, available at https://lindat.mff.cuni.cz/repository/xmlui/handle/11234/1-3683. The Lancaster Sensorimotor Norms are available at https://osf.io/7emr6/. Wordform data from the WOLEX database^105^ are not publicly available, but a subset can be made available upon request to the authors.

## Code availability

Code to reproduce our results is available via GitHub at http://github.com/Futrell/infolocality.
