# Supplementary material for: Linguistic structure from a bottleneck on sequential information processing
Source: Nat Hum Behav. 2025 Nov 24;10(3):589–600. doi: 10.1038/s41562-025-02336-w (PMC13017527; doi:10.1038/s41562-025-02336-w)
Supplement: Supplementary file 1 — Supplementary Sections A–G, Figs. 1–14, Tables 1 and 2 and References. [file 41562_2025_2336_MOESM1_ESM.pdf]

nature human behaviour

Supplementary information https://doi.org/10.1038/s41562-025-02336-w

Linguistic structure from a bottleneck on sequential information processing

In the format provided by the authors and unedited

Contents

A Basic formal results **1**
A.1 Forms of predictive information . . . 1
A.2 Predictive information for a finite-state source . . . 2
A.3 Length-2 languages . . . 3
A.4 Length-3 languages . . . 5
A.5 Length-T languages . . . 6
A.6 Predictive information for a random permutation . . . 8

B Sources over Two Features **10**

C Phonological Locality in 61 languages **14**

D NP Orders with Other Source Distributions **14**

E Correlation of Semantic Features **14**

F Phonotactic Results with Corpus Frequencies **14**

G Hierarchically-Structured Sources **14**
G.1 Varying Coupling Parameters in Tree Structures . . . 14
G.2 Sources Defined by PCFGs . . . 14

A Basic formal results

In the Main Text, based on numerical simulations, we claimed that codes that minimize predictive information tend to (1) factorize their source distribution into approximately independent components, and (2) express these components systematically in local parts of strings. Here we provide some elementary theorems about codes that minimize predictive information, which illustrate these generalizations. Although a full mathematical analysis of such codes is beyond the scope of the current work, the results below serve to establish their general behavior.

A.1 Forms of predictive information

In the Main Text, we claimed that the predictive information of a stochastic process can be thought of in terms of successive approximations to the entropy rate based on n-gram models with successively larger context size n. This has previously been shown by Crutchfield and Feldman (2003, Prop. 8), among others. Here we provide the same result by means of a different and more direct proof.

Consider a stationary stochastic process generating symbols labelled $\ldots, X_{t-1}, X_t, X_{t+1}, \ldots$ extending into the infinite past and future. Predictive information is defined as the limit of the mutual information of large blocks of M symbols before and N symbols after an arbitrary time index t:

$$E = \lim_{N\to\infty} \lim_{M\to\infty} \mathrm{I}[X_{t-M:t} : X_{t:t+N}], \tag{1}$$

where $X_{a:b} = X_a, \ldots, X_{b-1}$ represents a block of symbols X indexed by an exclusive range. We write Eq. 1 in shorthand as the mutual information between the infinite past $X_{<t}$ and infinite future $X_{\geq t}$ of the process,

$$E = \mathrm{I}[X_{<t} : X_{\geq t}]. \tag{2}$$

Now we can state the theorem relating predictive information to entropy rates derived from n-gram models.

Theorem 1. *The predictive information E can be written as*

$$E = \lim_{N \to \infty} \sum_{n=1}^{N} (h_n - h), \tag{3}$$

where h_n is the n-gram entropy rate

$$h_n = \mathrm{H}[X_t \mid X_{t-n+1:t}], \tag{4}$$

and h is the asymptotic entropy rate

$$h = \lim_{n \to \infty} h_n. \tag{5}$$

Proof. Invoking stationarity, we set $t = 1$ without loss of generality. Using the chain rule for mutual information, we rewrite the predictive information as a sum of conditional mutual informations:

$$E = \lim_{N \to \infty} \lim_{M \to \infty} \sum_{n=1}^{N} \mathrm{I}[X_{1-M:1} : X_n \mid X_{1:n}]. \tag{6}$$

Now we break each mutual information term into a difference of conditional entropies:

$$E = \lim_{N \to \infty} \lim_{M \to \infty} \sum_{n=1}^{N} \left(\mathrm{H}[X_n \mid X_{1:n}] - \mathrm{H}[X_n \mid X_{1-M:n}] \right). \tag{7}$$

Because conditioning reduces entropy, the terms $\mathrm{H}[X_n \mid X_{1-M:n}]$ (which are finite) converge monotonically downward in M, so we may swap the sum and the limit on M. Then, invoking stationarity again, we notice that the resulting two terms are the n-gram entropy rate and the asymptotic entropy rate:

$$E = \lim_{N \to \infty} \sum_{n=1}^{N} \left(\underbrace{\mathrm{H}[X_n \mid X_{1:n}]}_{n\text{-gram entropy rate}} - \underbrace{\lim_{M \to \infty} \mathrm{H}[X_n \mid X_{1-M:n}]}_{\text{asymptotic entropy rate}} \right) \tag{8}$$

$$= \lim_{N \to \infty} \sum_{n=1}^{N} (h_n - h), \tag{9}$$

$$\tag{10}$$

as claimed.

□

A.2 Predictive information for a finite-state source

The following result shows that predictive information is bounded at a constant when a language puts symbols in an order that respects the correlational structure of the source distribution, when the source distribution has the form of a Hidden Markov Model. On the other hand, we will show in Section A.6 that random orders have average predictive information that grows linearly with the sequence length.

Theorem 2. *Let $(S_t)_{t\geq 0}$ be a Hidden Markov Model (HMM) with finite state space $\mathcal{S}$ and finite emission alphabet $\mathcal{A}$ generating a bi-infinite stationary stochastic process $\ldots, X_{-1}, X_0, X_1, \ldots$. Let $L \in \mathbb{N}$, and consider the length-L language given by $X_1 \ldots X_L$. The predictive information is bounded independently of the sequence length L:*

$$\frac{1}{L}\sum_{i=1}^{L} \mathrm{I}\left[X_{1\ldots i} : X_{i+1\ldots L}\right] = O(1) \tag{11}$$

where $O(1)$ contains constants depending on the HMM but not L.

Proof. Let $s_i \in \mathcal{S}$ be the state of the HMM after generating $\ldots X_{i-2}X_{i-1}X_i$. Note that s_i is a random variable with $\mathrm{H}[s_i] \leq \log|\mathcal{S}|$. Further, $\mathrm{I}[X_{1\ldots i} : X_{i+1\ldots L}|s_i] = 0$. Hence, by the Data Processing Inequality, $\mathrm{I}[X_{1\ldots i} : X_{i+1\ldots L}] \leq \mathrm{H}[s_i] \leq \log|\mathcal{S}| = O(1)$ independently of L. □

A.3 Length-2 languages

We now analyze the most basic case of a code that minimizes predictive information, one in which every meaning is expressed in a string of length 2. We find that a code which minimizes predictive information in this setting performs Independent Components Analysis on the source distribution, with the two characters of the output string representing the two maximally independent factors of the source.

Let $\mathcal{M}$ be a set of meanings with source distribution p_M, Σ_1 and Σ_2 be disjoint sets of symbols, and $\mathcal{L}$ be a set of languages defined as bijections $L : \mathcal{M} \to \Sigma_1 \times \Sigma_2$. The predictive information of a language $E(L)$ is the predictive information of the stream of symbols generated by repeatedly sampling meanings from p_M, translating them to strings as $s = L(m)$, and concatenating the resulting strings with a delimiter $\# \notin \Sigma_1, \notin \Sigma_2$ between them.

Theorem 3. *Any language $L^* \in \mathcal{L}$ that achieves $E(L^*) = \min_{L\in\mathcal{L}} E(L)$ has the form*

$$L^*(m) = \ell_1(m) \cdot \ell_2(m), \tag{12}$$

where ℓ_i denotes some mapping $\ell_i : \mathcal{M} \to \Sigma_i$ and where the outputs from ℓ_1 and ℓ_2 have minimal mutual information:

$$\ell_1, \ell_2 = \arg\min \mathrm{I}[\ell_1(M) : \ell_2(M)], \tag{13}$$

with the minimization performed over all mappings $\mathcal{M} \to \Sigma_i$.

Proof. Because the languages have strings of length 2, we calculate predictive information as

$$E = h_1 + h_2 + h_3 - 3h, \tag{14}$$

up to length 3, accounting for the delimiter # attached after the end of the string. The entropy rate $h = \frac{1}{3}\mathrm{H}[M]$ is constant across all languages because they are all bijections, so we ignore the entropy rate going forward. Furthermore, there is no decrease in n-gram entropy rate for $n > 3$, so we have $h_3 = h$. Dropping all irrelevant constants, E is thus

$$E \sim h_1 + h_2. \tag{15}$$

Calculation of h_1: The unigram entropy rate is the entropy of the distribution over symbols generated by first sampling a time index t relative to the most recent delimiter, and then looking at

the symbol at that position. For a code of length T (including the delimiter to the right), this is

$$\begin{aligned}
h_1 &= -\sum_{t=1}^{T} p(t) \sum_{x\in\Sigma_t} p(X_t = x) \log p(t) p(X_t = x) \\
&= -\frac{1}{T}\sum_{t=1}^{T} \sum_{x\in\Sigma_t} p(X_t = x) \log \frac{1}{T} p(X_t = x) \\
&= -\frac{1}{T}\sum_{t=1}^{T} \log\frac{1}{T} - \frac{1}{T}\sum_{t=1}^{T} \sum_{x\in\Sigma_t} p(X_t = x) \log p(X_t = x) \\
&= \log T + \frac{1}{T}\sum_{t=1}^{T} \mathrm{H}[X_t],
\end{aligned}$$

that is, a constant reflecting how much information is contained in each symbol about its position in the string, plus the average entropy of symbols found in each position. Ignoring constants not affected by the choice of language L, in our case with $T = 3$ this is

$$h_1 \sim \mathrm{H}[X_1] + \mathrm{H}[X_2] + \underbrace{\cancel{\mathrm{H}[X_3]}}_{=0}, \tag{16}$$

where $\mathrm{H}[X_3] = 0$ because we always have $X_3 = \#$.

Calculation of h_2: The bigram entropy rate h_2 can be calculated following the same logic, yielding

$$h_2 \sim \underbrace{\mathrm{H}[X_1 \mid X_0]}_{=\mathrm{H}[X_1]} + \mathrm{H}[X_2 \mid X_1] + \underbrace{\cancel{\mathrm{H}[X_3 \mid X_2]}}_{=0}, \tag{17}$$

where $H[X_1 \mid X_0] = H[X_1]$ because X_0 is the left delimiter, which is uninformative about the value of X_1.

Putting these together and ignoring irrelevant constants yields

$$\begin{aligned}
E &\sim h_1 + h_2 && (18)\\
&\sim \mathrm{H}[X_1] + \mathrm{H}[X_2] + \mathrm{H}[X_1] + \mathrm{H}[X_2 \mid X_1] && (19)\\
&= \mathrm{H}[X_1] + \mathrm{H}[X_2 \mid X_1] + \mathrm{I}[X_1 : X_2] + \mathrm{H}[X_1] + \mathrm{H}[X_2 \mid X_1] && (20)\\
&= 2\,\mathrm{H}[X_1] + 2\,\mathrm{H}[X_2 \mid X_1] + \mathrm{I}[X_1 : X_2] && (21)\\
&= 2\,\mathrm{H}[X_1, X_2] + \mathrm{I}[X_1 : X_2] && (22)\\
&= 2\,\mathrm{H}[M] + \mathrm{I}[X_1 : X_2]. && (23)
\end{aligned}$$

Thus, we are left with

$$E \sim \mathrm{I}[X_1 : X_2], \tag{24}$$

where all remaining constants do not depend on the choice of language L. Without loss of generality, we can write $X_1 = \ell_1(M)$ and $X_2 = \ell_2(M)$ for any language L with the appropriate choice of the ℓ_1, ℓ_2, and thus we have that minimal predictive information is achieved by finding functions ℓ_1, ℓ_2 to minimize mutual information:

$$\arg\min \mathrm{I}[\ell_1(M) : \ell_2(M)]. \tag{25}$$

□

Remark. As predictive information is symmetrical with respect to time reversal, the solutions here are symmetric with respect to swapping ℓ_1 and ℓ_2.

Remark. The argument reveals that there is a degenerate solution when $|\Sigma_i| \geq |\mathcal{M}|$: you could encode the source M entirely with ℓ_i, with the other $\ell_{j \neq i}$ a constant function. In that case it is always possible to achieve $\mathrm{I}[\ell_1(M) : \ell_2(M)] = 0$. This result mirrors the claim from Nowak et al. (2000) that combinatorial communication requires that the number of available signals is less than the number of available meanings.

A.4 Length-3 languages

We now consider codes consisting of strings of length 3. We find that, in this setting, the *order* of the characters in the string is determined by information locality: the non-adjacent characters should be maximally uncorrelated, while the adjacent characters may be more correlated.

Now consider bijective languages $L : \mathcal{M} \to \Sigma_1 \times \Sigma_2 \times \Sigma_3$ producing strings of length 3, with the alphabets Σ_i all disjoint. Now we no longer have invariance with respect to interchanging the features ℓ_1, ℓ_2, ℓ_3: the order in which features are expressed now matters. Below, we show that languages which minimize E order these features so as to minimize the mutual information of the nonlocal features ℓ_1 and ℓ_3.

Theorem 4. *Any length-3 language $L^* \in \mathcal{L}$ that achieves $E(L^*) = \min_{L \in \mathcal{L}} E(L)$ has the form*

$$L^*(m) = \ell_1(m) \cdot \ell_2(m) \cdot \ell_3(m) \tag{26}$$

where the functions $\{\ell_i\}$ are ordered so that $\mathrm{I}[\ell_1(M) : \ell_3(M)]$ is minimal.

Proof. Dropping irrelevant constants in the length-3 case yields

$$E \sim \mathrm{I}[X_1 : X_2] + \mathrm{I}[X_2 : X_3] + 2\,\mathrm{I}[X_1 : X_3 \mid X_2]. \tag{27}$$

This expression can be written out and then rearranged as so:

$$\begin{aligned}
E &\sim \mathbb{E}\left[\ln \frac{p(X_1, X_2)}{p(X_1)p(X_2)}\right] + \mathbb{E}\left[\ln \frac{p(X_2, X_3)}{p(X_2)p(X_3)}\right] + 2\,\mathbb{E}\left[\ln \frac{p(X_1, X_2, X_3)p(X_2)}{p(X_1, X_2)p(X_2, X_3)}\right] && (28)\\
&= \mathbb{E}\left[\ln \frac{p(X_1, X_2, X_3)p(X_1, X_2, X_3)p(X_2)p(X_2)}{p(X_1)p(X_2)p(X_3)p(X_1, X_2)p(X_2, X_3)}\right] && (29)\\
&= \mathbb{E}\left[\ln \frac{p(X_1, X_2, X_3)}{p(X_1)p(X_2)p(X_3)}\right] + \mathbb{E}\left[\ln \frac{p(X_1, X_3 \mid X_2)}{p(X_1 \mid X_2)p(X_3 \mid X_2)}\right] && (30)\\
&= \mathrm{TC}[X_1 : X_2 : X_3] + \mathrm{I}[X_1 : X_3 \mid X_2] && (31)\\
&= \underbrace{\mathrm{TC}[X_1 : X_2 : X_3] - \mathrm{I}[X_1 : X_2 : X_3]}_{\text{Order-independent}} + \underbrace{\mathrm{I}[X_1 : X_3]}_{\text{Order-dependent}}, && (32)
\end{aligned}$$

where $\mathrm{TC}[\cdot : \cdots : \cdot]$ is total correlation (Watanabe, 1960) and $\mathrm{I}[\cdot : \cdots : \cdot]$ is multivariate mutual information (McGill, 1955). Both the TC term and the multivariate mutual information term are invariant to permutations, so the ordering of X_1, X_2, X_3 does not matter for them. The only term that depends on the order of symbols is $\mathrm{I}[X_1 : X_3]$. Thus any candidate optimal language L may be improved by permuting the functions ℓ_1, ℓ_2, ℓ_3 to minimize $I[\ell_1(M) : \ell_3(M)]$. □

Remark. The multivariate information term $\mathrm{I}[X_1 : X_2 : X_3]$ may be positive or negative. If it is positive, the situation is called redundancy. If it is negative, the situation is called synergy. The result above shows that codes with synergy among the three symbols X_1, X_2, X_3 are dispreferred, and codes with redundancy are preferred.

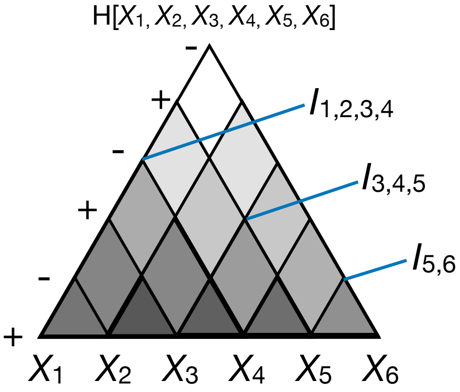

Figure 1: Schematic for coinformation in a set of 6 random variables, based on Bell (2003, Fig. 2). The joint entropy of $X_1, \ldots, X_6$ may be found by summing all the coinformations of all the strict subsets of these variables, weighted by the signs given to the left of the triangle. A few coinformations are highlighted. The true lattice of coinformations is a 6D Boolean hypercube; the figure shows a 2D reduction for visual clarity.

A.5 Length-T languages

Next, we consider the more general case of languages with utterance of a fixed length T, maintaining the setting where each position in the string has symbols from disjoint alphabets. We show that the predictive information for these languages may be expressed in terms of the coinformation lattice (Bell, 2003) among random variables corresponding to positions in the string. We find that predictive information is a function of the amount of coinformation in sets of variables and the *span size* of those sets, defined as the linear distance from the first character to the last character in the set. This gives a generalized form of information locality, where predictive information is low whenever any *set* of characters with high *synergy* are all close to each other.

Before stating the result, it is helpful to review the concept of coinformation. Consider a set of T random variables $X_1, \ldots, X_T$, and a set of indices such as, for example, $E = \{2, 3, 4\}$. Let X_E denote the random variables indexed by the set E, for example $X_E = \{X_2, X_3, X_4\}$. The **coinformation** among the random variables indexed by E is defined as

$$I_E = -\sum_{F \subseteq E} (-1)^{|F|} \, \mathrm{H}[X_F], \tag{33}$$

that is, the sum of entropies of all the subsets of X_E, weighted by 1 if the subset is of odd cardinality and -1 if the subset of is of even cardinality. For example, for $E = \{2, 3, 4\}$, the coinformation is

$$I_{2,3,4} = H[X_2] + H[X_3] + H[X_4] - H[X_2, X_3] - H[X_3, X_4] - H[X_2, X_4] + H[X_2, X_3, X_4]. \tag{34}$$

The coinformation generalizes entropy and mutual information. For a single variable, for example $E = \{1\}$, we recover the univariate entropy, $I_1 = \mathrm{H}[X_1]$. For two random variables, for example $E = \{1, 2\}$, we recover mutual information: $I_{1,2} = \mathrm{H}[X_1] + \mathrm{H}[X_2] - \mathrm{H}[X_1, X_2] = \mathrm{I}[X_1 : X_2]$. Coinformation for a set of variables is organized in a lattice structure, as illustrated in Figure A.5.

For $|E|$ odd (except for $|E| = 1$), coinformation can be negative, which corresponds to **synergy**, which happens for three variables when $I[X_1 : X_2 \mid X_3] > I[X_1 : X_2]$. Positive coinformation for an

odd number of variables corresponds to **redundancy**, which occurs when $I[X_1 : X_2 \mid X_3] < I[X_1 : X_2]$.

Coinformation may be interpreted as the amount of covariance among variables that cannot be detected from any strict subset of those variables. Synergy reflects a case when there is *more* such covariance, and redundancy reflects a case where there is less. Therefore, we can make the quantity of coinformation somewhat more interpretable by transforming it to **synergistic information** S, which makes synergy positive and redundancy negative:

$$S_E = (-1)^{|E|} I_E, \tag{35}$$

that is, to define synergistic information, we reverse the sign of coinformation for odd-numbered sets of variables. Synergistic information is positive when there is synergy among an odd-numbered set of variables, negative when there is redundancy, and positive when there is any coinformation among an even-numbered set of variables.

We can now state our result about predictive information in languages consisting of strings of length T.

Theorem 5. *For a language generating strings of fixed length T and disjoint alphabets for each string position, the predictive information E up to additive and multiplicative constants is*

$$E \sim \sum_{1 \leq a < \cdots < z \leq T} (z - a)\, S_{a,\ldots,z}, \tag{36}$$

where $S_{a,\ldots,z}$ is the synergistic information among the set of random variables $\{X_a, \ldots, X_z\}$ corresponding to characters at positions $a, \ldots, z$.

Proof. Up to additive and multiplicative constants, the predictive information in this language is

$$E \sim \sum_{t=1}^{T} \mathrm{I}[X_1, \ldots, X_t : X_{t+1}, \ldots, X_T] \tag{37}$$

$$= \sum_{t=1}^{T} \left(\mathrm{H}[X_1, \ldots, X_t] + \mathrm{H}[X_{t+1}, \ldots, X_T] - \mathrm{H}[X_1, \ldots, X_T] \right). \tag{38}$$

Next, we note that, inverting the definition of coinformation, we can write the entropy in a set of N variables as (Bell, 2003, p. 922)

$$\mathrm{H}[X_1, \ldots, X_N] = - \sum_{1 \leq a < \cdots < z \leq N} (-1)^{|a,\ldots,z|} I_{a,\ldots,z} \tag{39}$$

$$= - \sum_{1 \leq a < \cdots < z \leq N} S_{a,\ldots,z}, \tag{40}$$

where $a, \ldots, z$ is a set of indices defining a subset of the variables $X_1, \ldots, X_N$. We can use this to rewrite the predictive information in terms of synergistic information:

$$E \sim \sum_{t=1}^{T} \left(- \sum_{1 \leq a < \cdots < z \leq t} S_{a,\ldots,z} - \sum_{t+1 \leq a < \cdots < z \leq T} S_{a,\ldots,z} + \sum_{1 \leq a < \cdots < z \leq T} S_{a,\ldots,z} \right). \tag{41}$$

The question now is how many times we are adding in each synergistic information term $S_{a,\ldots,z}$ to get the total. We can imagine the whole expression as a sum over cut points t which split the string

into two parts, left and right. Within this sum, for each cut point, the last term adds in a synergistic information term $S_{a,\ldots,z}$ for each subset of indices $a, \ldots, z$, and the first two terms subtract all of the synergistic information terms whose indices are either entirely to the left of the cut point or to its right, leaving only those terms whose indices 'straddle' the cut, in the sense that at least one index is $\leq t$ and at least one index is $> t$. Thus, we can rewrite predictive information using an indicator variable for whether the set of indices $a, \ldots, z$ straddles the cut t. Then we count how often this indicator variable is equal to 1, yielding the result:

$$E \sim \sum_{t=1}^{T} \sum_{1\leq a<\cdots<z\leq T} 1_{a\leq t<z} S_{a,\ldots,z} \tag{42}$$

$$= \sum_{1\leq a<\cdots<z\leq T} \left(\sum_{t=1}^{T} 1_{a\leq t<z} \right) S_{a,\ldots,z} \tag{43}$$

$$= \sum_{1\leq a<\cdots<z\leq T} (z-a)\, S_{a,\ldots,z}. \tag{44}$$

□

Remark. It can easily be checked that the formula recovers Eq. 24, which was used in the proof of Theorem 2, for $T = 2$.

Remark. This result goes some way toward linking the hierarchical and well-nested structure of human language with predictive information. In fixed-length languages that minimize predictive information, *groups* of words or letters will tend to be close to each other as a function of how much they covary, in a way that is nested according to the structure of the coinformation lattice. Ill-nested configurations, in which groups of variables with high synergistic information are placed in such a way that other variables intervene, would contribute more to the predictive information, since the synergistic information in groups of variables is weighted by the span of those variables.

A.6 Predictive information for a random permutation

The following result shows that random orders have average predictive information that grows linearly with the sequence length. This is in contrast to our results from Section A.2 showing that, for finite-state processes, the predictive information is bounded independently of L.

Theorem 6. *Let $\ldots, X_{-1}, X_0, X_1, \ldots$ be a bi-infinite stationary process. Let $L \in \mathbb{N}$, and consider the length-L language given by $X_1 \ldots X_L$. Assume the process contains predictive information beyond its ergodic components (Dębowski, 2009), in the sense that:*

$$\inf_{\Delta>0} \mathrm{I}[X_w : X_{\ldots w-\Delta}] < \mathrm{I}[X_w : X_{\ldots,w-2,w-1}] \tag{45}$$

Consider the uniform distribution over bijections $\rho : [1, \ldots, L] \to [1, \ldots, L]$. Then

$$\mathbb{E}_\rho \left[\frac{1}{L} \sum_{i=1}^{L} \mathrm{I}\left[X_{\rho(1\ldots i)} : X_{\rho(i+1\ldots L)} \right] \right] = \Theta(L) \tag{46}$$

where the expectation describes an average over all bijections ρ, and constants in $\Theta(L)$ depend on the HMM but not L.

The intuition is that for any process with local statistical structure, beyond its ergodic components, permutations of the positions will tend to disrupt this local structure and create long-range dependencies.

Proof. The expectation is evidently $O(L)$; we need to show it is $\Omega(L)$. Define $A = \rho(1 \dots i)$, $B = \rho(i+1 \dots L)$. The proof idea is to focus attention on positions w where $w \in A$ but a contiguous sequence of positions to its left is in B. Such situations create opportunity for X_B to provide predictive information about X_A. Formally, for any $\Delta > 0$:

$$
\begin{aligned}
&\mathbb{E}[\mathrm{I}[X_A : X_B]] \\
&= \mathbb{E}\left[\sum_{w\in A} \mathrm{I}[X_w : X_{j\in B}|X_{j<w,j\in A}]\right] \\
&= \sum_{w=1}^{L} \mathbb{E}\left[1_{w\in A}\mathrm{I}[X_w : X_{j\in B}|X_{j<w,j\in A}]\right] \\
&\geq \sum_{w=1}^{L} \mathbb{E}\left[1_{w\in A}1_{[w-\Delta,w-1]\cap A=\emptyset}\mathrm{I}[X_w : X_{j\in B}|X_{j<w,j\in A}]\right] \\
&\geq \sum_{w=1}^{L} \mathbb{E}\left[1_{w\in A}1_{[w-\Delta,w-1]\cap A=\emptyset}\mathrm{I}[X_w : X_{[w-\Delta,w-1]}|X_{j<w,j\in A}]\right] \\
&= \sum_{w=1}^{L} \mathbb{E}\left[1_{w\in A}1_{[w-\Delta,w-1]\cap A=\emptyset}\mathrm{I}[X_w : X_{[w-\Delta,w-1]}|X_{j<w-\Delta,j\in A}]\right] \\
&= \sum_{w=1}^{L} p_\rho(w\in A; [w-\Delta, w-1]\cap A = \emptyset)\mathbb{E}\left[\mathrm{I}[X_w : X_{[w-\Delta,w-1]}|X_{j<w-\Delta,j\in A}]|w\in A, 1_{[w-\Delta,w-1]\cap A=\emptyset}\right]
\end{aligned}
$$

We now need to show that, for large Δ,

$$
\mathrm{I}[X_i : X_{[w-\Delta,w-1]}|X_{j<w-\Delta,j\in A}] \tag{47}
$$

is bounded away from 0 uniformly over A. Consider[1]

$$
\begin{aligned}
&\mathrm{I}[X_w : X_{[w-\Delta,w-1]} \mid X_{j<w-\Delta,j\in A}] \\
&= \mathrm{I}[X_w : X_{[w-\Delta,w-1]}] - \mathrm{I}[X_w : X_{j<w-\Delta,j\in A}] + \mathrm{I}[X_w : X_{j<w-\Delta,j\in A} \mid X_{[w-\Delta,w-1]}] \\
&\geq \mathrm{I}[X_w : X_{[w-\Delta,w-1]}] - \mathrm{I}[X_w : X_{j<w-\Delta,j\in A}] \\
&\geq \mathrm{I}[X_w : X_{[w-\Delta,w-1]}] - \mathrm{I}[X_w : X_{j<w-\Delta}]
\end{aligned}
$$

When $\Delta \to \infty$, the first term converges to $\mathrm{I}[X_w|X_{\dots w-2,w-1}]$. By assumption, the difference between this and the second term is strictly greater than zero. Overall, this shows (47) is bounded strictly

[1] Reflecting the general identity

$$
\begin{aligned}
\mathrm{I}[A : B \mid C] &= H[A \mid C] - H[A \mid C, B] \\
&= H[A] - H[A \mid B] - H[A] + H[A \mid C] + H[A \mid B] - H[A \mid B, C] \\
&= \mathrm{I}[A : B] - \mathrm{I}[A : C] + \mathrm{I}[A : C \mid B]
\end{aligned}
$$

away from zero independently of A, for some sufficiently large Δ which we henceforth fix for the given HMM, independently of L. Let $C > 0$ be this lower bound for (47).

It remains to understand why, assuming $|A|$ and $|B|$ are sufficiently large, $\mathbb{E}[\mathrm{I}[X_A : X_B]]$ is $\Omega(L)$. Given the Δ we have fixed,

$$p_\rho(w \in A; [w-\Delta, w-1] \cap A = \emptyset) \geq D > 0 \tag{48}$$

for a constant D independent of w, for L sufficiently large, when $0.1L < |A| < 0.9L$. For, in this case, we have

$$\begin{aligned}
&p_\rho(w \in A; [w-\Delta, w-1] \cap A = \emptyset) \\
&= p_\rho(w \in A) \cdot \prod_{j=1}^{\Delta} p_\rho(w-j \in B | w \in A, w-1 \in B, \dots, w-j+1 \in B) \\
&= \underbrace{p(\rho(w) \leq i)}_{=\frac{i}{L}} \cdot \prod_{j=1}^{\Delta} \underbrace{p\left(\rho(w-j) > i | \rho(w) \leq i, \rho(w-1) > i, \dots, \rho(w-j+1) > i\right)}_{=\frac{L-i-j+1}{L-j}} \\
&\geq \frac{i}{L} \cdot \left(\frac{L-i-\Delta}{L}\right)^{\Delta} \\
&\geq \frac{1}{10} \left(\frac{0.1L-\Delta}{L}\right)^{\Delta} \\
&\geq \frac{1}{10} \cdot \frac{1}{20^{\Delta}} =: D
\end{aligned}$$

where the last step holds when $L > 20\Delta$. Taken together,

$$\mathbb{E}[\mathrm{I}[X_A : X_B]] \geq L \cdot D \cdot C = \Omega(L) \tag{49}$$

when $0.1L < |A| < 0.9L$. The claim follows. □

We note that one can strengthen the proof to provide a high-probability bound, showing that *most* permutations ρ satisfy such linear scaling. The reason is that a random permutation, when $|A|$ and $|B|$ are both large, is very likely to satisfy the event described in (48) on a constant fraction of positions w.

B Sources over Two Features

Simulation results in the main text are based on distributions of the form

$$p(M) = p(M_1) \times p(M_2, M_3) \tag{50}$$

for varying levels of correlation between the binary random variables M_2 and M_3. The main result is that when M_2 and M_3 have lower mutual information, a systematic code for these features minimizes predictive information, but as mutual information increases, a holistic code is more preferred. Here we complement these results with a more in-depth study of a source distribution over two features of the form $p(M) = p(M_1, M_2)$ for binary random variables M_1 and M_2, looking at a grid of possible distributions over 4 outcomes. This comprehensive approach allows us to examine the effects of the marginal probabilities for M_1 and M_2, as well as the effects of different *kinds* of correlations between features on the relative preference for systematic vs. holistic codes.

Outcome	Corr. Source	Anticorr. Source	Systematic	cnot(1,2)	cnot(2,1)
00	3/8	0	ac	ac	ac
01	1/8	1/4	ad	ad	bd
10	1/8	1/4	bc	bd	bc
11	3/8	1/2	ad	bc	ad

Table 1: Some possible sources and codes for the two binary random variables M_1, M_2. The correlated source has Pearson's $r = 1/2$. The anticorrelated source has $r = -1/3$.

The main result is shown in Figure 2, which shows predictive information for all possible mappings from the four outcomes of M to strings in $\{\mathtt{a}, \mathtt{b}\} \times \{\mathtt{c}, \mathtt{d}\}$. The rows indicate different marginal probabilities for $p(M_1 = 1)$, the columns indicate different marginal probabilities for $p(M_2 = 1)$, and the x axis indicates the Pearson correlation between M_1 and M_2. The Pearson correlation is necessary to make sense of the pattern here, because two kinds of correlation can induce mutual information between M_1 and M_2: a *positive* correlation between the most probable outcomes and a *negative* correlation, as shown in Table 1. In the positive correlation case, the features M_1 and M_2 are effectively 'fused'—at maximal correlation, there is actually only one feature here, as we always have $M_1 = M_2$. In the negative correlation case, it is as if one of the four outcomes has been effectively removed from the probability distribution.

There are two conclusions to be drawn from Figure 2 beyond the conclusions in the main text. First, the level of preference for systematicity in the low-correlation case depends on the marginal distributions being imbalanced: at $p(M_1) = p(M_2) = \frac{1}{2}$, even when there is zero correlation between the features, the holistic code is just as good as the systematic code. This makes sense because for a uniform distribution over 4 outcomes, there is no reason to favor any one factorization over another. However, as the marginals become more imbalanced (moving downward or to the right in the figure), the systematic code becomes better in the low-correlation range. For these imbalanced marginals, there are generally two red lines to be seen in the figure, corresponding to the two possible classes of non-systematic codes for the source: cnot$(1, 2)$ and cnot$(2, 1)$, which differ in which feature is used as the control bit to flip the other one.

The second conclusion to be drawn from Figure 2 is that there is different behavior for positive and negative feature correlations when the marginals for M_1 and M_2 are both imbalanced. In particular, in the lower right corner, the systematic code is sometimes better than the holistic code when there is a negative correlation. This happens because, in the negatively correlated source, the systematic code allows the appearance of individual symbols to be correlated with the overall probability of the string: for example, in the systematic code for the negatively correlated source in Table 1, high-frequency strings always have d, and a only appears in low-frequency strings. The result is that the unigram entropy is minimized by the systematic code for such a source.

Figure 3 shows predictive information for codes as a function of mutual information between random variables M_1 and M_2, with the negatively-correlated sources separated out and indicated with a dotted line. We see that the preference for holistic codes as a function of mutual information is weaker for the negatively correlated sources, and also that these sources cannot achieve mutual information as high as the positively correlated ones.

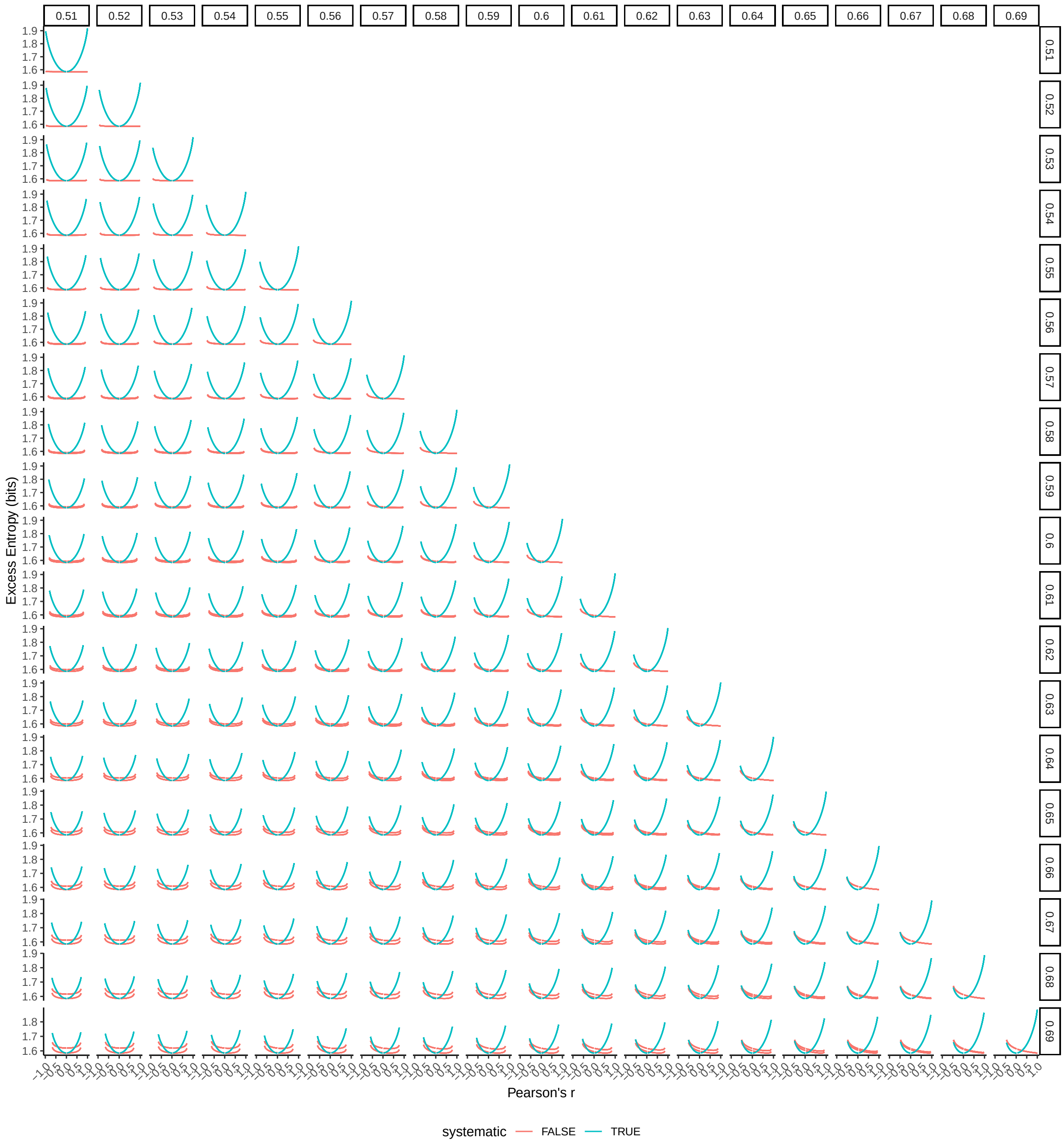

Figure 2: Predictive information (labelled as excess entropy) of length-2 codes for a grid over the simplex of possible sources over two binary random variables, $p(M) = p(M_1, M_2)$. Rows show the marginal probability $p(M_1 = 1)$. Columns show the marginal probability $p(M_2 = 1)$. The x axis shows the Pearson correlation between M_1 and M_2.

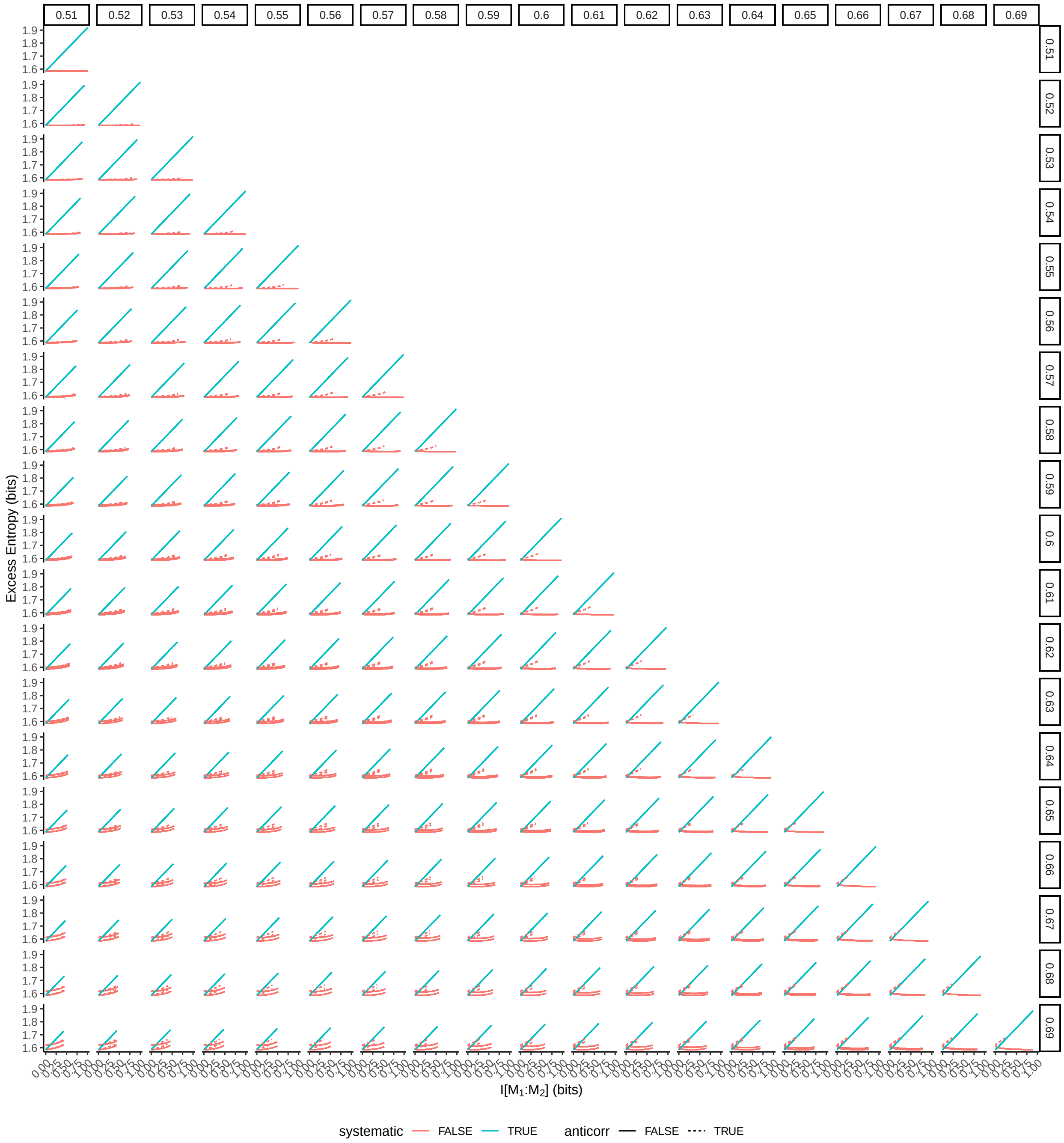

Figure 3: Predictive information (labelled as excess entropy) of codes for a grid over the simplex of possible sources over two binary random variables as in Figure 2, but now by mutual information instead of Pearson correlation. Dotted lines indicate codes for sources whose Pearson correlation is negative.

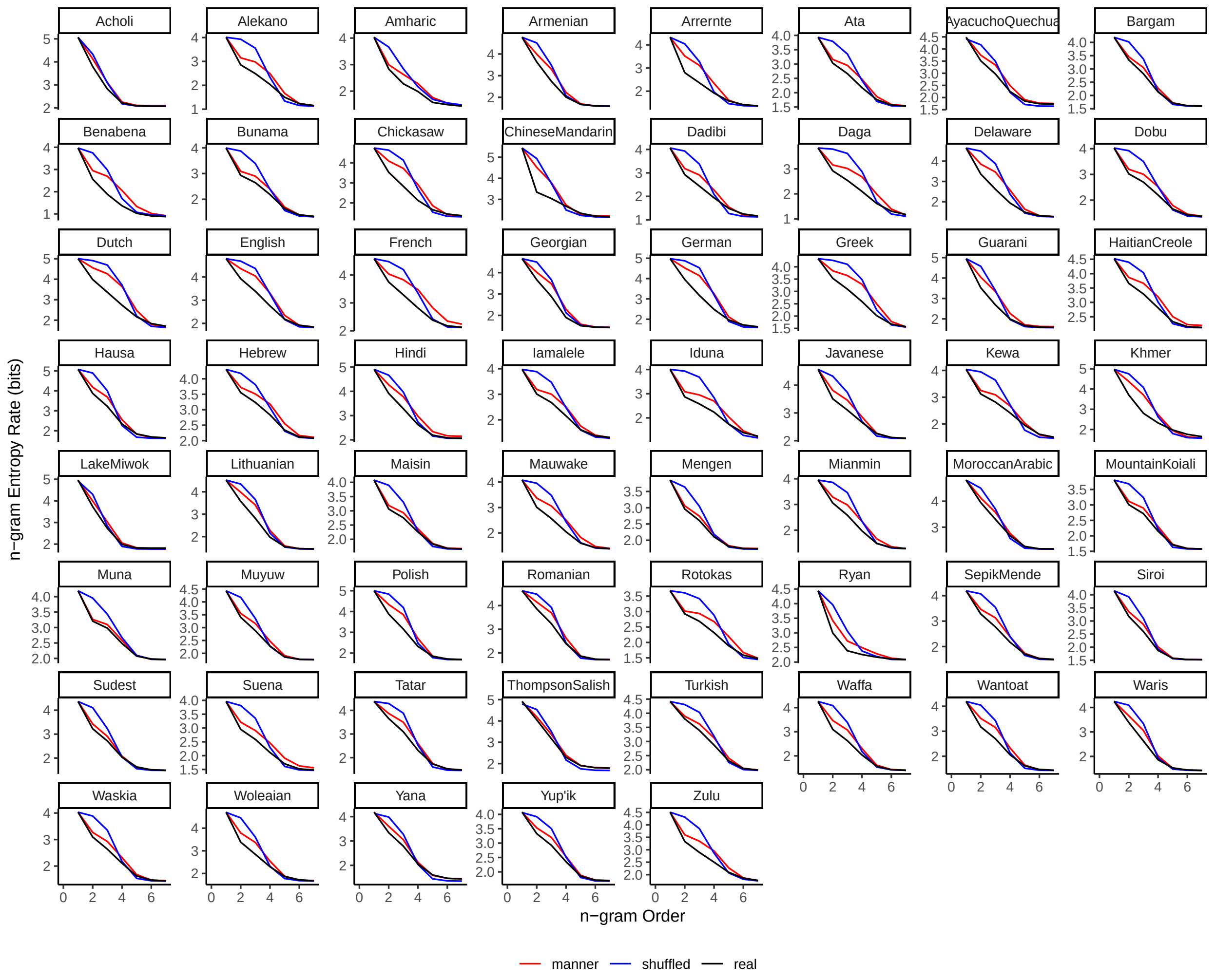

Figure 4: Calculation of predictive information for all 61 languages in the WOLEX database, for the attested forms (black), a deterministic shuffle that preserves manner of articulation (red), and a general deterministic shuffle (blue).

C Phonological Locality in 61 languages

Figure 4 shows the calculation of predictive information for all 61 languages in the WOLEX database, for the real languages compared against two baselines generated by applying deterministic shuffling functions to the attested forms. Table 2 shows the calculated predictive information values.

D NP Orders with Other Source Distributions

The noun phrase ordering results in the main text were derived using a source distribution over NPs estimated from the German Universal Dependencies corpus. Here we show results using other naturalistic source distributions, from corpora of Spanish (Figure 5), English (Figure 6), Czech (Figure 7), Icelandic (Figure 8), and Latin (Figure 9). We also show results using the artificial source developed by Mansfield and Kemp (2023) to study NP order in Figure 10.

**Language**	**Real**	**Manner**	**Shuffled**
Acholi	5.64	6.17	6.39
Alekano	7.42	8.79	9.60
Amharic	5.62	6.67	7.42
Armenian	6.91	8.04	8.60
Arrernte	6.34	8.17	8.37
Ata	5.98	6.76	7.59
Ayacucho Quechua	6.49	7.23	7.82
Bargam	6.26	6.74	7.45
Benabena	6.57	8.92	9.33
Bunama	6.94	7.59	8.69
Chickasaw	8.60	10.80	11.30
Dadibi	7.43	8.43	9.15
Daga	7.99	9.70	10.60
Delaware	7.88	9.97	10.60
Dobu	6.99	7.86	8.91
Dutch	9.38	11.90	12.40
English	6.91	8.34	8.65
French	6.35	7.78	8.50
Georgian	7.52	8.83	9.38
German	8.83	11.20	11.60
Greek	8.10	10.20	10.90
Guarani	7.00	8.21	8.66
Haitian Creole	6.05	6.82	7.63
Hausa	8.35	9.21	9.83
Hebrew	5.96	6.83	7.33
Hindi	6.64	7.56	8.12
Iamalele	7.21	8.10	9.09
Iduna	8.02	9.38	10.60
Javanese	5.72	6.57	7.08
Kewa	7.22	7.92	8.82
Khmer	8.30	10.00	10.50
Lake Miwok	6.20	6.75	6.87
Lithuanian	7.24	8.54	9.00
Maisin	5.72	6.07	7.03
Mandarin Chinese	6.06	7.65	8.05
Mauwake	6.53	8.05	8.79
Mengen	4.66	4.95	5.85
Mianmin	6.80	8.01	8.83
Moroccan Arabic	6.20	6.71	6.99
Mountain Koiali	5.55	5.99	6.72
Muna	5.13	5.46	6.53
Muyuw	6.12	6.79	7.36
Polish	7.85	9.35	9.90
Romanian	7.44	8.37	8.67
Rotokas	6.49	7.49	8.41
Ryan	3.87	5.02	5.63
Sepik Mende	6.77	7.48	8.47
Siroi	5.79	6.37	7.06
Sudest	6.59	6.96	7.90
Suena	6.08	6.89	7.74
Tatar	7.96	8.82	9.39
Thompson Salish	7.49	7.87	8.38
Turkish	7.00	7.66	8.37
Waffa	6.64	7.74	8.49
Wantoat	6.63	7.69	8.17
Waris	6.55	7.39	7.98
Waskia	6.40	7.09	7.85
Woleaian	6.83	7.92	8.57
Yana	6.82	7.38	8.13
Yup'ik	6.09	6.75	7.43
Zulu	6.79	8.12	9.00

Table 2: Predictive information values (in bits) for 61 languages of the WOLEX sample, visualized in Figure 4. 'Real' is the predictive information of the attested wordforms. 'Manner' is for wordforms shuffled while preserving manner. 'Shuffled' is for wordforms shuffled without regard for manner.

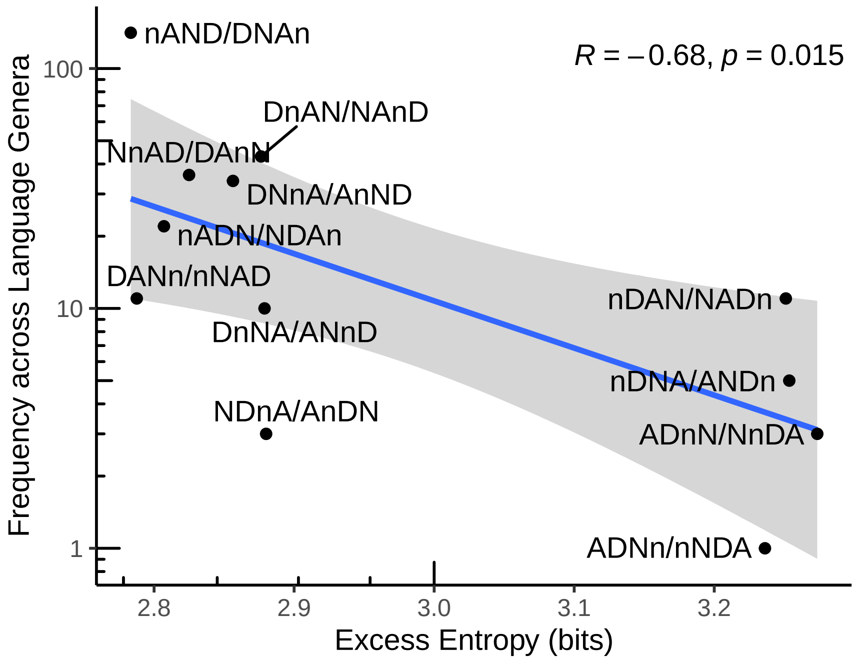

Figure 5: Typology frequencies of NP orders by predictive information estimated using the **Spanish UD source** (Mariona Taulé and Recasens, 2008). Lines and statistics as in the figure in the main text.

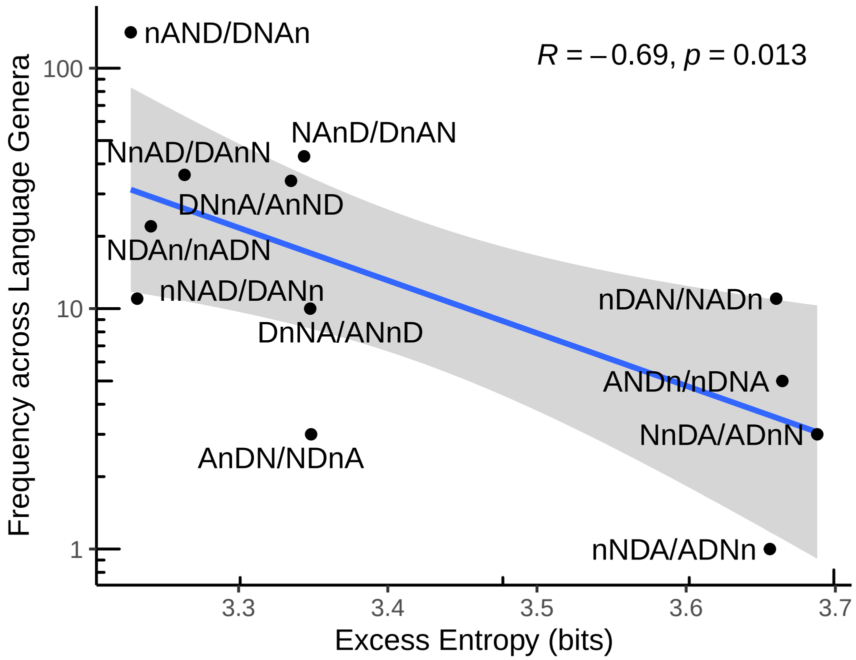

Figure 6: Typology frequencies of NP orders by predictive information estimated using the **English UD source** (Zeldes, 2017).

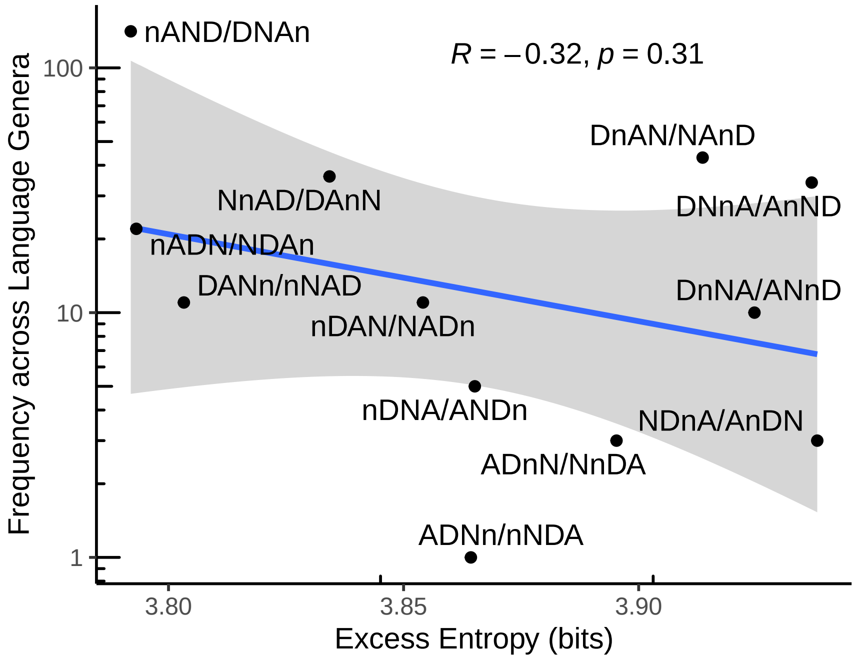

Figure 7: Typology frequencies of NP orders by predictive information estimated using the **Czech UD source** (Hladká et al., 2008). We believe the weaker correlation here is due to the rarity of determiners in the Czech corpus.

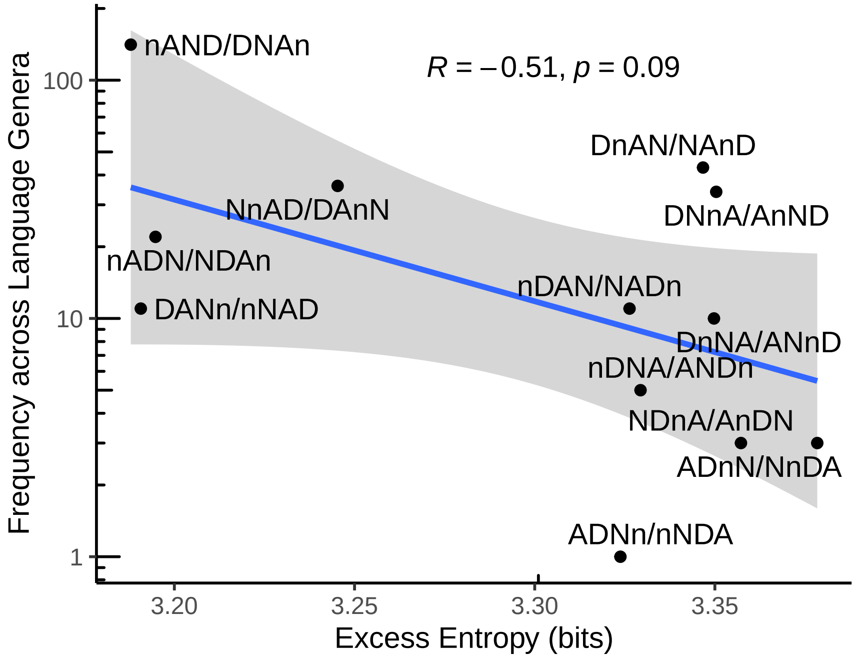

Figure 8: Typology frequencies of NP orders by predictive information estimated using the **Icelandic UD source** (Arnardóttir et al., 2020).

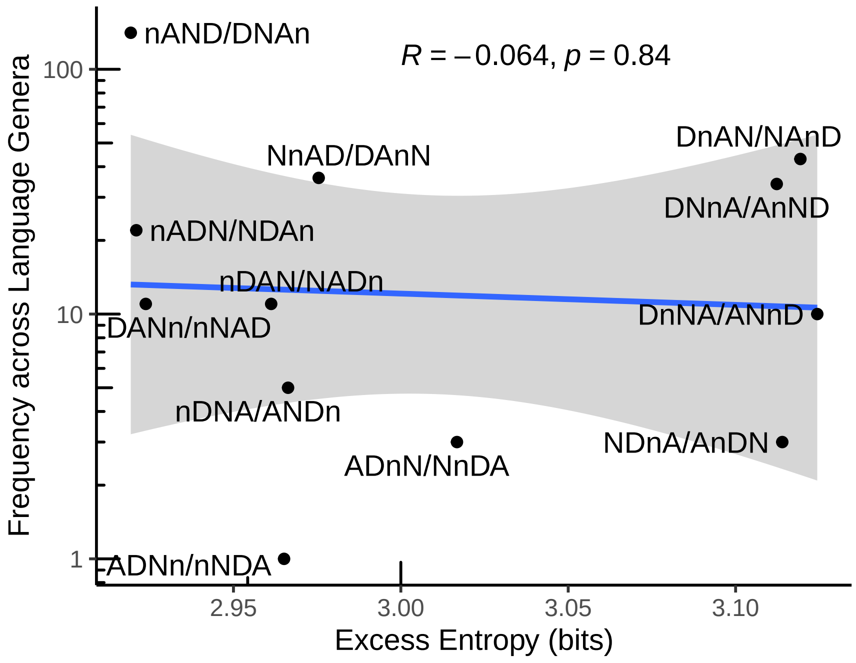

Figure 9: Typology frequencies of NP orders by predictive information estimated using the **Latin UD source** (based on all Latin UD corpora). As the text genre for this corpus is highly unusual (consisting of over 1000 years' worth of text, much of it poetry or written by non-native speakers), we believe that the distribution of NPs in this corpus is not representative of the 'true' source distribution over NP meanings.

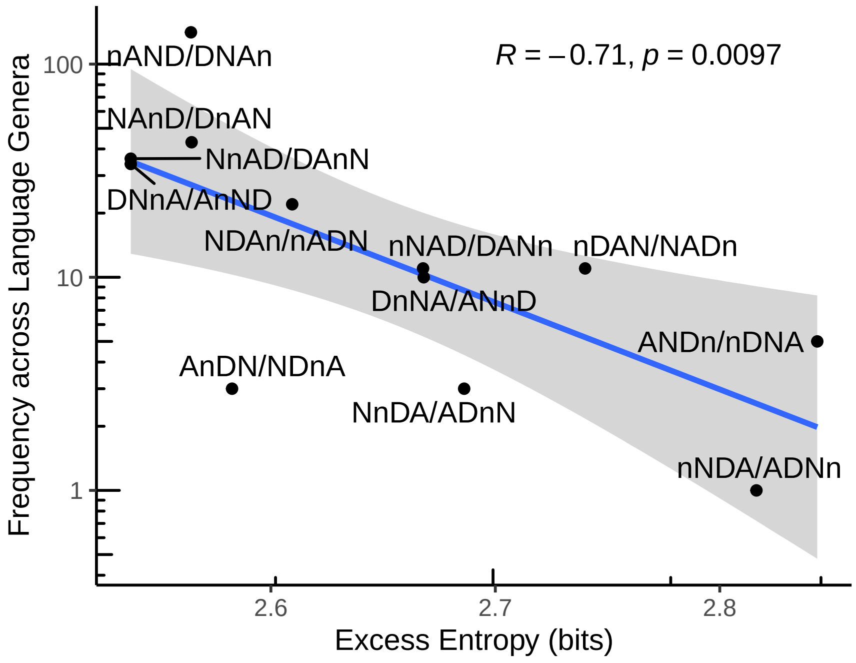

Figure 10: Typology frequencies of NP orders by predictive information estimated using the artificial **MK23 source** (Mansfield and Kemp, 2023).

E Correlation of Semantic Features

In Figure 11 we present results of the study on correlation of semantic features, but using the semantic feature norms from the Glasgow Word Norms (Scott et al., 2017), which rate words for features such as dominance, valence, and arousal. Features are binarized and their frequencies and pairwise MIs are calculated as in the main text. Results are similar to the main text: the across-morpheme and across-word features largely have lower mutual information than within-morpheme and within-word features.

F Phonotactic Results with Corpus Frequencies

Recall that our analysis of phonotactics assumed a uniform distribution over forms. This is because the phonological forms, as listed in the WOLEX database, cannot straightforwardly be matched to corpus data. However, for four languages (Dutch, English, French, and German), WOLEX provides orthographic forms. Using these, we derived corpus frequencies from the full Wikipedia texts in these languages. We applied simple Laplace smoothing at $\alpha = 1$. Results as shown in Figure 12 closely agree with those derived under a uniform distribution.

G Hierarchically-Structured Sources

G.1 Varying Coupling Parameters in Tree Structures

We created further sources by keeping the tree structure from Main Paper, Figure 2F, but varying the parameters $\alpha, \beta, \gamma \in [0, 1]$ randomly subject to the constraint $4\alpha < 2\beta < \gamma$. We created 70 random samples. Results, shown in Figure 13, reproduce the pattern from Main Paper, Figure 2F.

G.2 Sources Defined by PCFGs

We constructed probabilistic context-free grammars (PCFGs) defined by 5 terminals and 5 nonterminals. For each nonterminal a, we considered the 100 possible binary productions $a \to bc$ where b, c are terminals or nonterminals. For each nonterminal, we defined a distribution over these 100 possible productions $a \to bc$ by defining

$$p(a \to bc) \propto \exp(T p_{a \to bc}), \tag{51}$$

where $T > 0$ is an inverse temperature parameter and each $p_{a \to bc} \in [0, 1]$ is a random number (cf. DeGiuli, 2019). The probabilities are normalized to sum up to one for each left-hand side a. The inverse temperature parameter controls the variability in the probabilities of different productions; higher values result in a sparser source.

We then enumerated all 5^6 strings of length 6 over the given nonterminals, and used the CKY algorithm to compute the probabilities of all of these strings under the given PCFG. This defines a source over all strings of length 6.

At inverse temperatures $T = 1, 2, 3, 4, 10, 20$ we sampled 10 PCFGs each, and compared the predictive information of the language given by the PCFG (systematic and local), deterministic permutations of the 6 positions (systematic and nonlocal), and 360 randomly chosen shuffles of the mapping between forms and probabilities (neither local nor systematic).

Results (Figure 14) show that local orderings usually achieve lower predictive information. Nonsystematic codes have much higher predictive information, very closely concentrated around values clearly separated from the systematic codes.

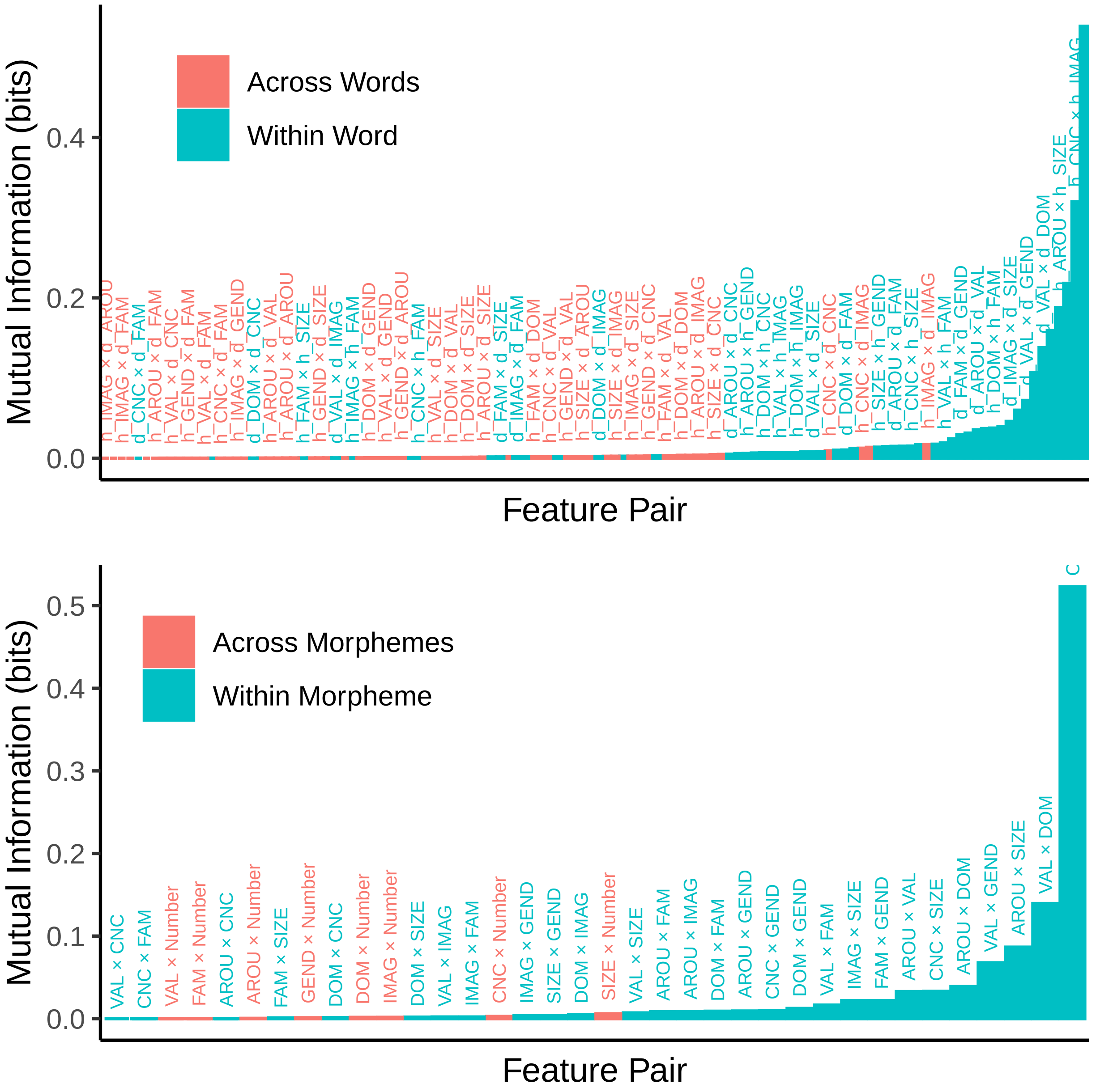

Figure 11: (A) Pairwise mutual information of semantic features from the Glasgow Word Norms (Scott et al., 2017) across words and within words, for pairs of verbs and objects in Universal Dependencies English corpora. (B) Pairwise mutual information of the Glasgow Word Norms along with a number feature indicated by plural morphology. The across-word and across-morpheme features have generally lower MI than the within-word and within-morpheme features.

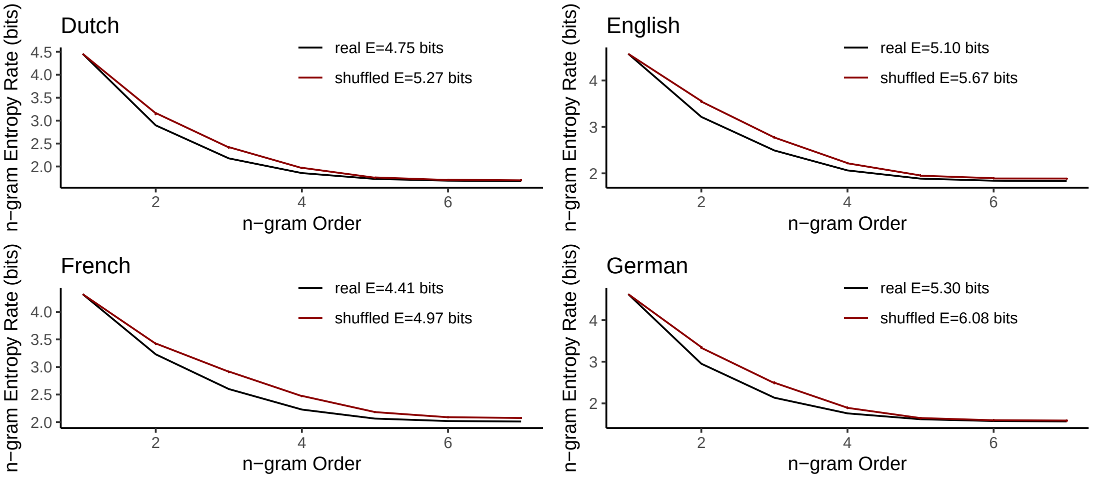

Figure 12: Calculation of predictive information using corpus frequencies, for the 4 languages in the WOLEX database for which orthographic forms are available in WOLEX. We show the attested forms (black) and a deterministic shuffle that preserves manner of articulation (red). Results match those found with uniform distributions.

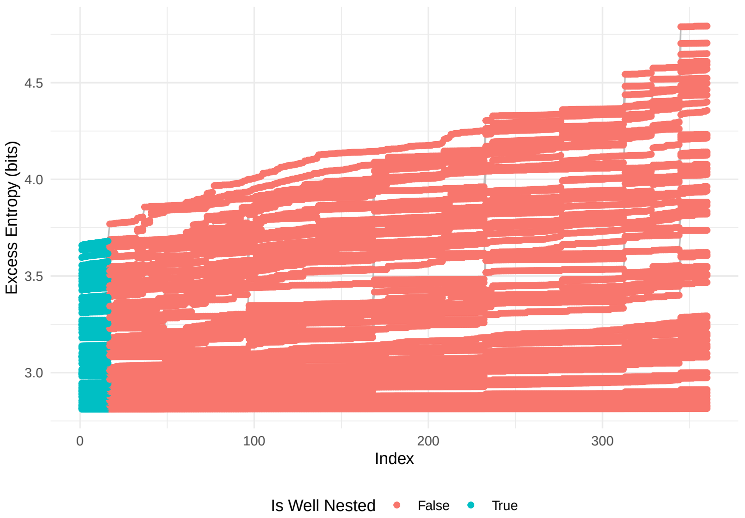

Figure 13: Results for 70 sampled combinations of coupling parameters for the tree structure in Main Paper, Figure 2F. Across samples, well-nested orderings achieve lower predictive information than non-well-nested orderings.

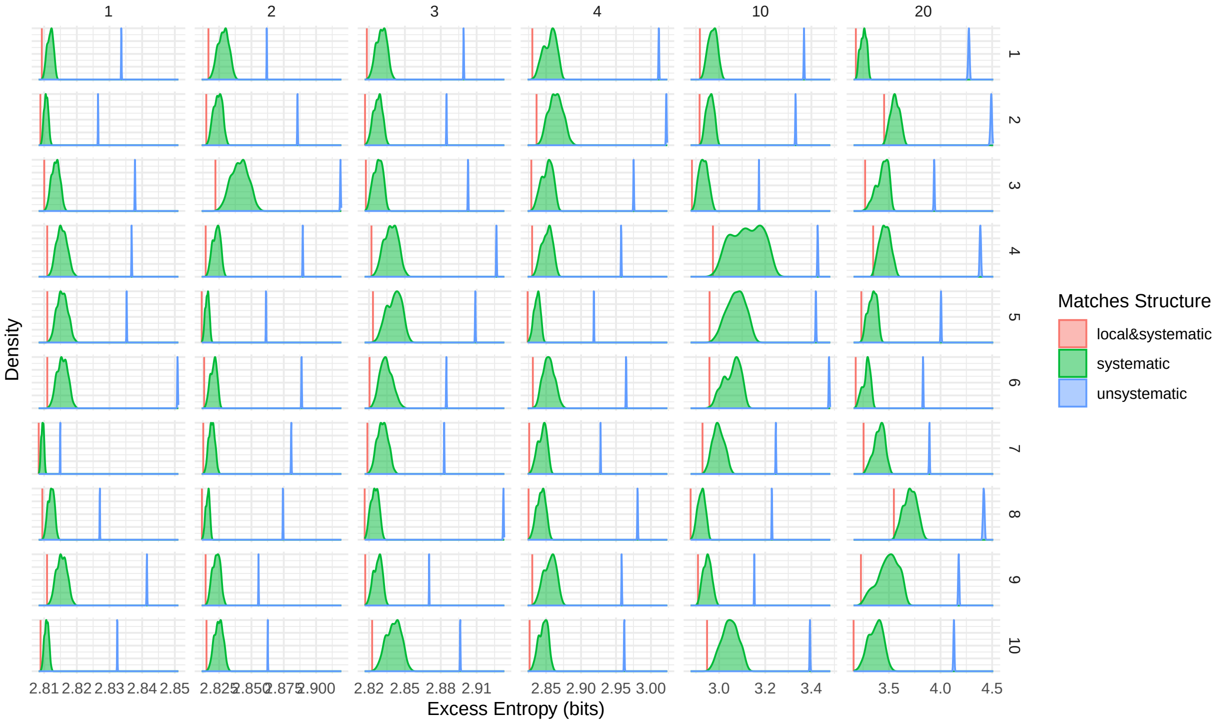

Figure 14: Distribution of predictive information, for 10 randomly constructed PCFG sources for length-6 strings over 5 symbols, at six different inverse temperature parameters (T in (51)). We compare the local and systematic code given the PCFG (red) with the systematic codes given by the deterministic shuffles of the six positions (green), and an equal number (360, up to reversal) of unsystematic codes given by shuffles of the mapping between forms and probabilities (blue). Local and systematic codes tend to achieve lower predictive information than other systematic codes. Unsystematic codes strongly concentrate at substantially higher predictive information.

References

Arnardóttir, Þ., Hafsteinsson, H., Sigurðsson, E. F., Bjarnadóttir, K., Ingason, A. K., Jónsdóttir, H., and Steingrímsson, S. (2020). A Universal Dependencies conversion pipeline for a Penn-format constituency treebank. In *Proceedings of the Fourth Workshop on Universal Dependencies (UDW 2020)*, pages 16–25, Barcelona, Spain (Online). Association for Computational Linguistics.

Bell, A. J. (2003). The co-information lattice. In *Proceedings of the 4th International Symposium on Independent Component Analysis and Blind Signal Separation (ICA2003)*, pages 921–926.

Crutchfield, J. P. and Feldman, D. P. (2003). Regularities unseen, randomness observed: Levels of entropy convergence. *Chaos: An Interdisciplinary Journal of Nonlinear Science*, 13(1):25–54.

Dębowski, Ł. (2009). A general definition of conditional information and its application to ergodic decomposition. *Statistics & Probability Letters*, 79(9):1260–1268.

DeGiuli, E. (2019). Random language model. *Physical Review Letters*, 122(12):128301.

Hladká, B., Hajic, J., Hana, J., Hlavácová, J., Mírovskỳ, J., and Raab, J. (2008). The Czech academic corpus 2.0 guide. *The Prague Bulletin of Mathematical Linguistics*, 89:41.

Mansfield, J. and Kemp, C. (2023). The emergence of grammatical structure from inter-predictability. *PsyArXiv*.

Mariona Taulé, M. A. M. and Recasens, M. (2008). AnCora: Multilevel annotated corpora for Catalan and Spanish. In Calzolari, N., Choukri, K., Maegaard, B., Mariani, J., Odijk, J., Piperidis, S., and Tapias, D., editors, *Proceedings of the Sixth International Conference on Language Resources and Evaluation (LREC'08)*, Marrakech, Morocco. European Language Resources Association (ELRA). http://www.lrec-conf.org/proceedings/lrec2008/.

McGill, W. J. (1955). Multivariate information transmission. *IEEE Transactions on Information Theory*, 4(4):93–111.

Nowak, M. A., Plotkin, J. B., and Jansen, V. A. A. (2000). The evolution of syntactic communication. *Nature*, 404(6777):495–498.

Scott, G. G., Keitel, A., Becirspahic, M., O'Donnell, P. J., and Sereno, S. C. (2017). The Glasgow Norms: Ratings of 5,500 words on 9 scales.

Watanabe, S. (1960). Information theoretical analysis of multivariate correlation. *IBM Journal of Research and Development*, 4(1):66–82.

Zeldes, A. (2017). The GUM Corpus: Creating multilayer resources in the classroom. *Language Resources and Evaluation*, 51(3):581–612.
